# Oxidative DNA Damage, Inflammatory Signature, and Altered Erythrocytes Properties in Diamond-Blackfan Anemia

**DOI:** 10.3390/ijms21249652

**Published:** 2020-12-17

**Authors:** Katarina Kapralova, Ondrej Jahoda, Pavla Koralkova, Jan Gursky, Lucie Lanikova, Dagmar Pospisilova, Vladimir Divoky, Monika Horvathova

**Affiliations:** 1 Department of Biology, Faculty of Medicine and Dentistry Palacky University, 775 15 Olomouc, Czech Republic; katka.kapralova@gmail.com (K.K.); ondrej.jahoda@upol.cz (O.J.); pavla.koralkova@gmail.com (P.K.); jan.gursky@upol.cz (J.G.); vladimir.divoky@upol.cz (V.D.); 2 Institute of Molecular Genetics of the Czech Academy of Sciences, 142 20 Prague, Czech Republic; lucie.lanikova@img.cas.cz; 3 Department of Pediatrics, Palacky University and University Hospital Olomouc, 779 00 Olomouc, Czech Republic; dagmar.pospisilova@fnol.cz

**Keywords:** Diamond-Blackfan anemia, reactive oxygen species, 8-oxoguanine, DNA damage response, inflammatory cytokines, erythrocyte lifespan

## Abstract

Molecular pathophysiology of Diamond-Blackfan anemia (DBA) involves disrupted erythroid-lineage proliferation, differentiation and apoptosis; with the activation of p53 considered as a key component. Recently, oxidative stress was proposed to play an important role in DBA pathophysiology as well. CRISPR/Cas9-created Rpl5- and Rps19-deficient murine erythroleukemia (MEL) cells and DBA patients’ samples were used to evaluate proinflammatory cytokines, oxidative stress, DNA damage and DNA damage response. We demonstrated that the antioxidant defense capacity of Rp-mutant cells is insufficient to meet the greater reactive oxygen species (ROS) production which leads to oxidative DNA damage, cellular senescence and activation of DNA damage response signaling in the developing erythroblasts and altered characteristics of mature erythrocytes. We also showed that the disturbed balance between ROS formation and antioxidant defense is accompanied by the upregulation of proinflammatory cytokines. Finally, the alterations detected in the membrane of DBA erythrocytes may cause their enhanced recognition and destruction by reticuloendothelial macrophages, especially during infections. We propose that the extent of oxidative stress and the ability to activate antioxidant defense systems may contribute to high heterogeneity of clinical symptoms and response to therapy observed in DBA patients.

## 1. Introduction

Diamond-Blackfan anemia (DBA) is a rare congenital bone marrow failure syndrome characterized by severe anemia, erythroblastopenia, congenital malformations and predisposition to cancer. DBA is mostly related to pathogenic variants in ribosomal protein (RP) genes, which cause their haploinsufficiency and a consequent defect in ribosome biogenesis [1]. The most frequent ones are mutations in *RPS19* (25%) and *RPL5* (7%) [1]. The molecular pathophysiology of DBA is attributed to both p53-dependent and p53-independent mechanisms, which lead to the proapoptotic and hypoproliferative phenotype of erythroid cells [2]. Research in the last few years suggested an important role of oxidative stress in DBA. Specifically, increased production of reactive oxygen species (ROS) was observed in shRNA model reproducing DBA, and in cultured primary cells from DBA patients [3]. Moreover, antioxidant treatment was reported to reduce p53 stabilization in RP-deficient cells in vitro [4]. Therefore, it has been suggested that the hypoproliferative phenotype in RP-mutant diseases is associated with increased oxidative stress and DNA damage [2]. Consistently, the activation of DNA damage response (DDR) pathway was detected in RPS19-deficient zebrafish and human fetal liver cells [5].

In certain types of congenital anemia, increased ROS levels are associated with a shortened life span of red blood cells (RBCs) [6,7]. It has also been shown that the accelerated clearance of RBCs in response to oxidative stress is attributed not only to excessive hemolysis, but also to the induction of a programmed cell death of erythrocytes, called eryptosis [6,8]. Although elevated levels of reduced glutathione (GSH), an essential antioxidant, were detected in DBA erythrocytes [9]; the extent of oxidative stress and its possible impact on RBCs’ properties has not been investigated in detail.

Here, we aimed to examine the extent of oxidative stress in DBA using patients’ samples and Rpl5- and Rps19-deficient cellular models. We observed that the antioxidant defense in Rp-mutant cells is insufficient to cope with increased ROS production, leading to oxidative DNA damage, apoptosis or senescence of erythroid precursors and altered characteristics of mature erythrocytes. This state was accompanied by the induction of proinflammatory cytokines which appeared to play an important role in DBA pathobiology. Despite some known differences in the molecular mechanisms involved in RPL5- and RPS19-mutant DBA [1,3], the above-mentioned features are common to both ribosomal protein deficiencies with a certain degree of variation in the intensity of the pathological phenotype.

## 2. Results

### 2.1. Characterization of Patients’ Cohort and Assessment of RBCs’ Oxidative Stress

The cohort consisted of Czech and Slovak DBA patients (*n* = 24) [10]. The majority of them (22/24) harbored a mutation in one of the RPs (Table 1). Only erythrocytes of patients in disease remission (*n* = 12) or hematologically stable on corticosteroids (*n* = 7) were used for the assessment of RBCs’ oxidative stress and enzyme activities. Patients with the most severe anemia receiving repeated erythrocyte transfusions were not included because the donor erythrocytes, likely representing the major fraction in the sample, would yield skewed results. Nevertheless, bone marrow samples from these patients (*n* = 5, P20–P24) were used for immunohistochemical (IHC) staining. The list of tests performed using samples from individual patients can be found in Table 1.

**Figure 1:**
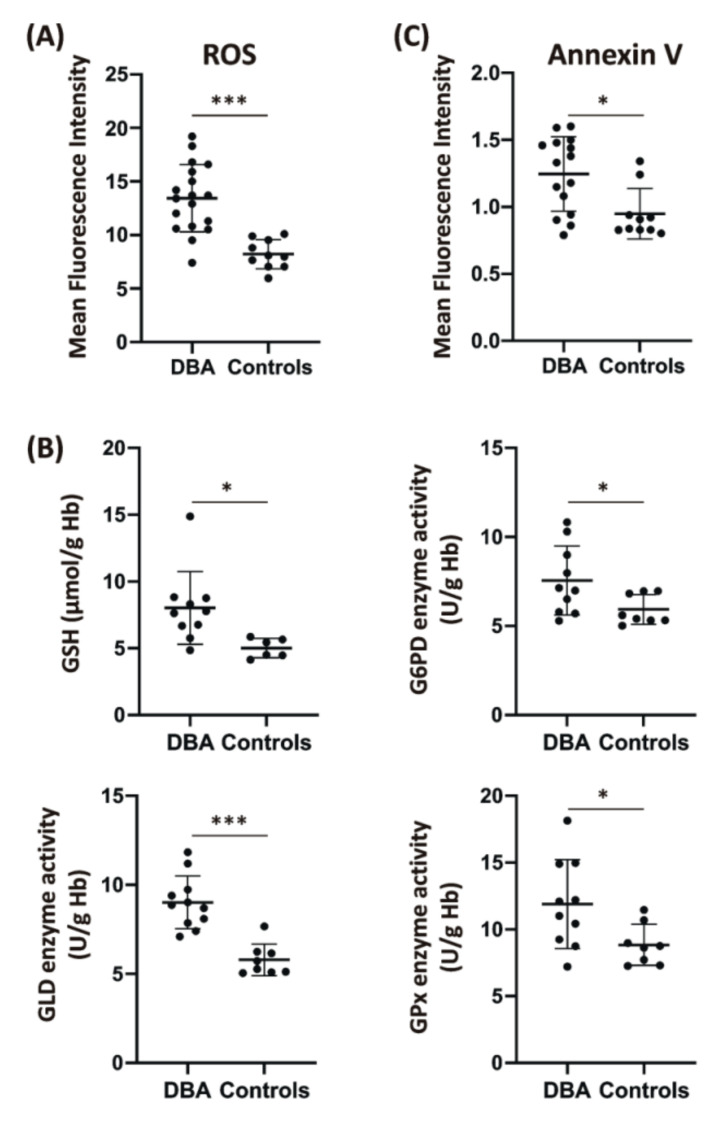
Oxidative stress, antioxidant defense, and Annexin V binding in Diamond-Blackfan anemia (DBA) patients’ erythrocytes. (**A**) Significantly elevated intracellular reactive oxygen species (ROS) content in DBA erythrocytes (*n* = 18) compared to control erythrocytes (*n* = 10). For a positive control, erythrocytes were exposed to 2 mM hydrogen peroxide (H_2_O_2_) for 10 min before 2′,7′-dichlorofluorescein diacetate (DCF-DA)-labeling (positive control mean fluorescence intensity (MFI): 230 ± 83). (**B**) Significantly increased reduced glutathione (GSH) levels (given as µmol/g hemoglobin—Hb) and enzymes activities of glucose-6-phosphate dehydrogenase (G6PD), gluconolactone dehydrogenase (GLD) and glutathione peroxidase (GPx) (expressed as U/g Hb) in DBA erythrocytes (*n* = at least 10) compared to controls (*n* = at least 6). Specific enzyme activity was calculated using the Lambert-Beer law. (**C**) Enhanced Annexin V binding to the membrane of DBA erythrocytes (*n* = 15) compared to controls (*n* = 10). Individual values in Panels (**A**–**C**) are presented in a dot plot depicting mean with error bars showing standard deviations (SDs). Graphs were created and *p* values calculated using GraphPad Prism 8 Software (La Jolla, CA, USA, www.graphpad.com); * *p* < 0.05, *** *p* < 0.001.

Initially, peripheral blood erythrocytes were stained with 2′,7′-dichlorofluorescein diacetate (DCF-DA) and the DCF-dependent fluorescence intensity, which is proportional to ROS levels, was measured by flow cytometry. As shown in Figure 1A, significantly increased levels of ROS were observed in DBA erythrocytes (mean fluorescence intensity—MFI: 13.2 ± 3.2) compared to control erythrocytes (MFI: 7.8 ± 1.2), confirming the presence of oxidative stress. Subsequently, the levels of reduced glutathione (GSH) and the activities of enzymes of the pentose phosphate pathway and antioxidant defense were assessed in leukocyte- and platelet-free erythrocytes lysates (Figure 1B). GSH levels, expressed as µmol/g hemoglobin (Hb), were significantly elevated in DBA erythrocytes (7.7 ± 2.73) compared to controls (4.98 ± 0.66). Concomitantly, significantly increased activities (U/g Hb) of glucose-6-phosphate dehydrogenase (G6PD; 7.07 ± 1.94 vs. 5.51 ± 0.83), gluconolactone dehydrogenase (GLD; 8.87 ± 1.48 vs. 5.5 ± 0.9), and glutathione peroxidase (GPx; 11.55 ± 3.33 vs. 8.69 ± 1.53) in DBA erythrocytes, compared to control erythrocytes, showed induction of antioxidant defense.

Thereafter, exposure of phosphatidylserines (PS) on the erythrocyte membrane, as one of the markers of eryptosis [6], was assessed by flow cytometric analysis of Annexin V binding. DBA erythrocytes showed significantly increased Annexin V binding (MFI: 1.30 ± 0.34) when compared to control erythrocytes (MFI: 0.95 ± 0.21) (Figure 1C). This confirms increased PS exposure on the membrane of DBA erythrocytes and suggests that excessive ROS production exceeds the capacity of scavenging mechanisms in DBA erythrocytes, resulting in erythrocyte membrane alterations which may cause their enhanced recognition and destruction by reticuloendothelial macrophages [7,11].

### 2.2. Creation and Validation of DBA Cellular Model

To investigate the extent of oxidative damage in DBA erythroid lineage in more detail, CRISPR/Cas9 technology was used to create Rpl5- and Rps19-deficient murine erythroleukemia (MEL) cells (a detailed description of the procedure can be found in Supplementary Materials and Figure S1A,B). *RPL5* and *RPS19* are the two most frequently mutated and studied genes associated with DBA [1]. Two clones for each gene with reduced levels of Rpl5 and Rps19 (Figure S1C,D) mimicking the Rp haploinsufficiency showed by DBA patients, were selected for further analyses. MEL cells are arrested at the proerythroblast stage and can be chemically induced to erythroid differentiation [12]. Both uninduced cells and cells induced to erythroid differentiation by dimethylsulfoxid (DMSO) for 96 h were evaluated in this study.

First, the typical DBA characteristics were observed in the created Rpl5- and Rps19-deficient cells. This included significantly decreased proliferation capacity, as measured by thiazolyl blue tetrazolium bromide (MTT) test at 48 and 72 h of cultivation (Figure S2A), and higher percentage of nucleated cells undergoing apoptosis detected by terminal deoxynucleotidyl transferase dUTP nick end labeling (TUNEL) assay on cytospin slides (Figure S2B) when compared to control cells. Immunoblot analysis of p53 phosphorylation revealed increased level of p53 activation in Rpl5- and Rps19-deficient cells (Figure S2C). More profound alterations in all above-mentioned assays were detected for Rpl5-deficient cells compared to Rps19-deficient cells, in agreement with a more severe phenotype associated with RPL5 deficiency [3]. Upon induction of erythroid differentiation, the fold-change in the mRNA expression of GATA-binding factor 1 (*Gata1*), a critical regulator of erythroblast maturation, was substantially lower in Rpl5- and Rps19-deficient cells (1.2-fold and 1.7-fold, respectively) when compared to control cells (2.4-fold) (Figure S3). Altogether, these data are consistent with impaired erythroid differentiation [1,13,14] and augmented erythroblasts apoptosis [1,14] that we previously reported in the bone marrow of DBA patients [15].

### 2.3. Oxidative DNA Damage in DBA Cellular Model

**Figure 2:**
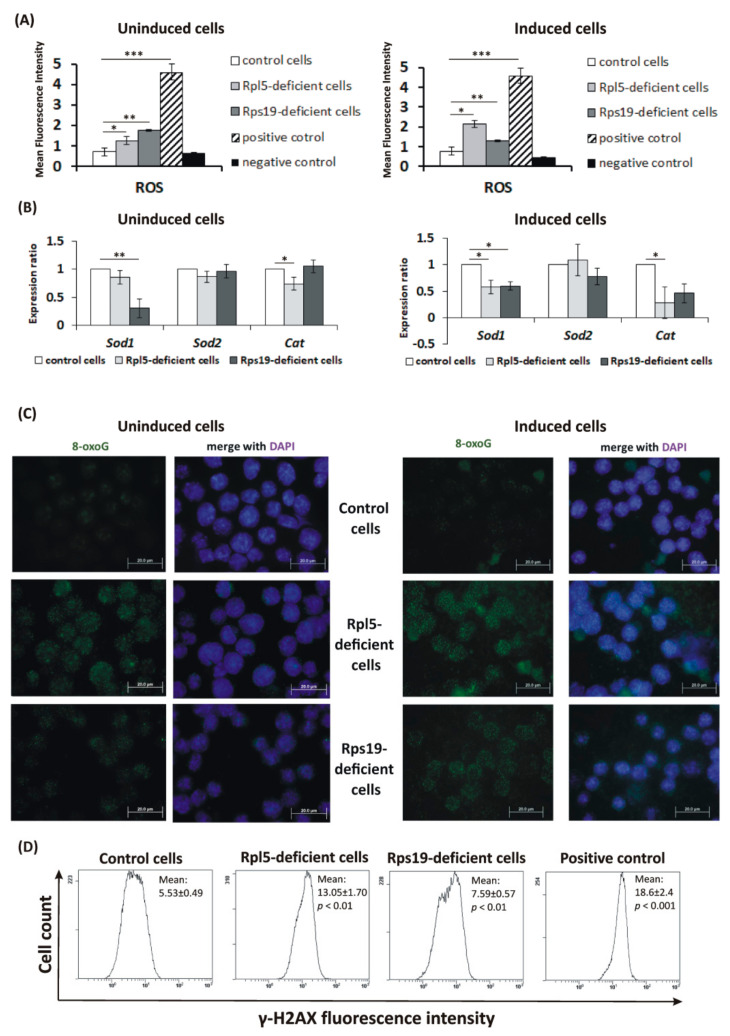
Oxidative stress and DNA damage in Rpl5- and Rps19-deficient murine erythroleukemia (MEL) cells. (**A**) Elevated reactive oxygen species (ROS) levels in uninduced and induced Rpl5- and Rps19-deficient cells compared to control cells. Values are given as mean ± standard deviation (SD). The results represent the mean of four independent experiments. *p* values were calculated using the Student *t*-test; * *p* < 0.05, ** *p* < 0.01, *** *p* < 0.001. For a positive control, MEL cells were exposed to 200 μM tert-butyl hydroperoxide (kit component) 1 h before staining with the ROS detection reagent; negative control—unstained cells. (**B**) Decreased relative mRNA expression of superoxide dismutase 1 (*Sod1)* and catalase (*Cat)* (normalized to beta-actin) in uninduced and induced Rpl5- and Rps19-deficient cells compared to controls. *p* values were calculated using the REST© 2020 software (Technical University Munich, Germany): * *p* < 0.05, ** *p* < 0.01; T bars designate standard error of the mean (SEM). (**C**) Higher nuclear and perinuclear 8-oxoguanine (8-oxoG) positivity (green color) for Rpl5- and Rps19-deficient cells compared to control cells. Cell nuclei were counterstained with 4′,6-diamidino-2-phenylindole dihydrochloride (DAPI, blue color). A positive control for 8-oxoG staining is shown in Figure S5. Immunostained cells were analyzed with an Olympus BX 51 fluorescence microscope (Olympus), original magnification 1000×. Digital images were acquired with an Olympus DP 50 camera driven by DP controller software (provided by Olympus, Tokyo, Japan). Images were cropped, assembled, and labeled using Adobe Photoshop software (Adobe Systems, San Jose, CA, USA). (**D**) Increased phosphorylation of Ser-139 residue of the histone variant H2AX (γ-H2AX) in uninduced Rpl5- and Rps19-deficient cells compared to controls. Values are given as mean ± SDs. Representative histograms of the assay, repeated 3 times, are shown. *p* values were calculated using the Student *t*-test; ** *p* < 0.01, *** *p* < 0.001. Positive control: γ-rays irradiated MEL cells (mean fluorescence intensity—MFI: 18.6 ± 2.4). MFI for unstained control cells: 0.86 ± 0.13.

After the basic characterization of MEL clones, CellROX Green reagent was used to determine intracellular ROS content. Significantly increased ROS levels were detected in uninduced Rpl5-deficient (MFI: 1.24 ± 0.16) and Rps19-deficient (MFI: 1.75 ± 0.39) cells when compared to control cells (MFI: 0.71 ± 0.11), and also in both Rp-deficient cells induced to erythroid differentiation (Rpl5-deficient, MFI: 2.14 ± 0.35; Rps19-deficient, MFI: 1.30 ± 0.17; controls, MFI: 0.76 ± 0.40) (Figure 2A). To assess the antioxidant capacity of Rpl5- and Rps19-deficient cells, expression analysis of critical antioxidant enzyme: superoxide dismutase 1 (SOD1), SOD2, and catalase (CAT) was evaluated. As shown in Figure 2B, predominantly *Sod1* and *Cat* mRNA are downregulated in Rpl5- and Rps19-deficient cells compared to control cells; the difference is more pronounced in Rp-deficient cells induced to erythroid differentiation. Further analysis of antioxidant defense enzymes activities (G6PD, GLD, and GPx) revealed that none of the enzymes showed upregulated activity (Figure S4) which would balance the elevated ROS levels in Rpl5- and Rps19-deficient cells. Altogether, this suggests insufficient capacity of Rpl5- and Rps19-deficient cells to cope with the greater ROS accumulation.

In order to assess the damaging effect of elevated ROS on DNA, immunocytochemical (ICC) analysis of an oxidative DNA lesion 8-oxoguanine (8-oxoG) [16] was performed. This ICC staining revealed elevated 8-oxoG levels on cytospin slides of Rpl5- and Rps19-deficient cells than in control cells (Figure 2C). As a result of ROS production, DNA damage may occur, resulting in DDR, which includes phosphorylation of Ser-139 residue of the histone variant H2AX (γ-H2AX) [16]. Indeed, a significantly elevated γ-H2AX signal, detected by increased fluorescence intensity, was observed for uninduced Rpl5-deficient cells (MFI: 13.05 ± 1.7) compared to control cells (MFI: 5.53 ± 0.49) (Figure 2D); the MFI for γ-irradiated control cells that served as a positive control was 18.6 ± 2.4. On the other hand, γ-H2AX in Rps19-deficient cells was less significantly induced (MFI: 7.59 ± 0.57), suggesting a lesser extent of DNA damage. Because γ-H2AX levels dramatically increase during late-stage erythropoiesis [17], MEL cells induced to erythroid differentiation were not evaluated.

### 2.4. Activation of DDR Signaling and DNA Repair in DBA Cellular Model

**Figure 3:**
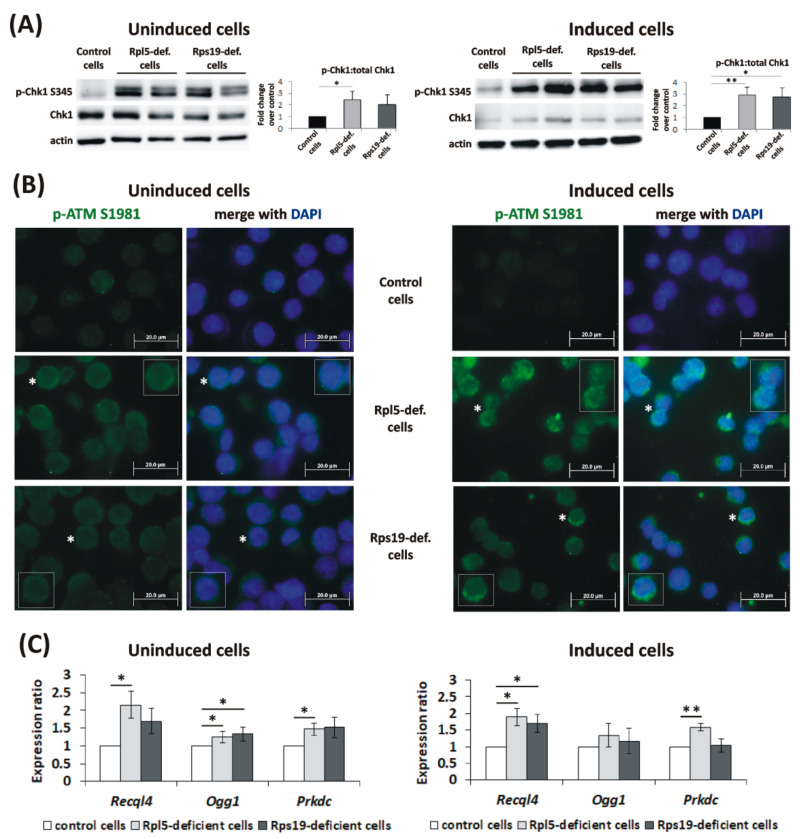
DNA damage response (DDR) signaling and DNA repair in Rpl5- and Rps19-deficient murine erythroleukemia (MEL) cells. (**A**) Increased levels of Chk1 phosphorylation at S345 (p-Chk1 S345) were detected in uninduced and induced Rpl5- and Rps19-deficient cells than in controls. A representative immunoblot is shown. p-Chk1 was normalized to total Chk1 protein using the G:BOX-CHEMI-XX9 imaging system (Syngene, Cambridge, UK). Data in the bar graph showed a fold change in p-Chk1:total Chk1 ratio over control cells and are expressed as means ± standard errors of the mean (SEM) from 3 independent experiments. * *p* < 0.05 and ** p  <  0.01 versus control cells. (**B**) Higher cytoplasmic positivity for phosphorylated ATM (p-ATM at S1981, green color) was observed in uninduced and induced Rpl5- and Rps19-deficient cells compared to control cells. Induced Rpl5-deficient cells showed, in addition, nuclear p-ATM S1981 staining. Cells nuclei were counterstained with 4′,6-diamidino-2-phenylindole dihydrochloride (DAPI) (blue color). The asterisks indicate cells shown in the insets. Immunostained cells were analyzed with an Olympus BX 51 fluorescence microscope (Olympus), original magnification 1000×. Digital images were acquired with an Olympus DP 50 camera driven by DP controller software (provided by Olympus, Tokyo, Japan). Images were cropped, assembled, and labeled using Adobe Photoshop software (Adobe Systems, San Jose, CA, USA). (**C**) Markers of DNA repair (*Recql4*, *Ogg1*, and *Prkdc*) showed variable increase in mRNA expression (normalized to beta-actin) in uninduced and induced Rpl5- and Rps19-deficient cells compared to controls. *p* values were calculated using the REST© 2020 software (Technical University Munich, Germany): * *p* < 0.05, ** *p* < 0.01; T bars designate SEM.

To assess the consequent activation of DDR signaling in response to the observed DNA damage, phosphorylation of Chk1 (p-Chk1) [18] and ATM (p-ATM) [19] was analyzed by immunoblot analysis and ICC staining, respectively. Increased levels of p-Chk1 at S345 in Rpl5-deficient cells and to a lesser degree also in Rps19-deficient cells, compared to controls (Figure 3A), confirmed the induction of ATR-Chk1 signaling. On the other hand, the staining against p-ATM at S1981 revealed predominantly cytoplasmic positivity in Rpl5- and Rps19-deficient cells (Figure 3B), which likely reflects ATM response to ROS [20]. Faint nuclear staining, reflecting a certain threshold level of endogenous DNA damage, was detected only in Rpl5- deficient cells (Figure 3B). These data are in agreement with differences in γ-H2AX fluorescence intensity observed between Rpl5- and Rps19-deficient cells.

Subsequently, selected mRNA expression markers of activated DNA repair were tested. This included an ATP-dependent DNA helicase Q4 (encoded by the *Recql4* gene) involved in homologous recombination (HR), nonhomologous end joining (NHEJ), nucleotide excision repair (NER) and base excision repair (BER) [21,22]; 8-Oxoguanine glycosylase (encoded by the *Ogg1* gene), a marker of BER responding to the presence of 8-oxoG lesions [23]; and a DNA-dependent protein kinase, catalytic subunit (encoded by the *Prkdc* gene) participating in NHEJ [24]. Uninduced Rpl5-deficient cells showed statistically increased mRNA expression of all analyzed markers (Figure 3C), with *Recql4* and *Prkdc* also being significantly upregulated in induced Rpl5-deficient cells. For Rps19-deficient cells, only the expression of *Ogg1* mRNA in uninduced cells and *Recql4* mRNA in cells induced to erythroid differentiation was statistically increased compared to the controls (Figure 3C). The expression of DNA repair genes appears to be more strongly induced in Rpl5-deficient cells than in Rps19-deficient cells, corresponding to a higher rate of DNA damage in Rpl5-deficient cells. As all analyzed markers are reported to be Gata1 targets [25], the degree of their upregulation may be restrained by reduced *Gata1* expression in induced Rpl5- and Rps19-deficient cells (Figure S3) and consequently limit the activation of DNA repair.

### 2.5. Elevated Inflammatory Cytokines and Senescence in DBA Cellular Model

**Figure 4:**
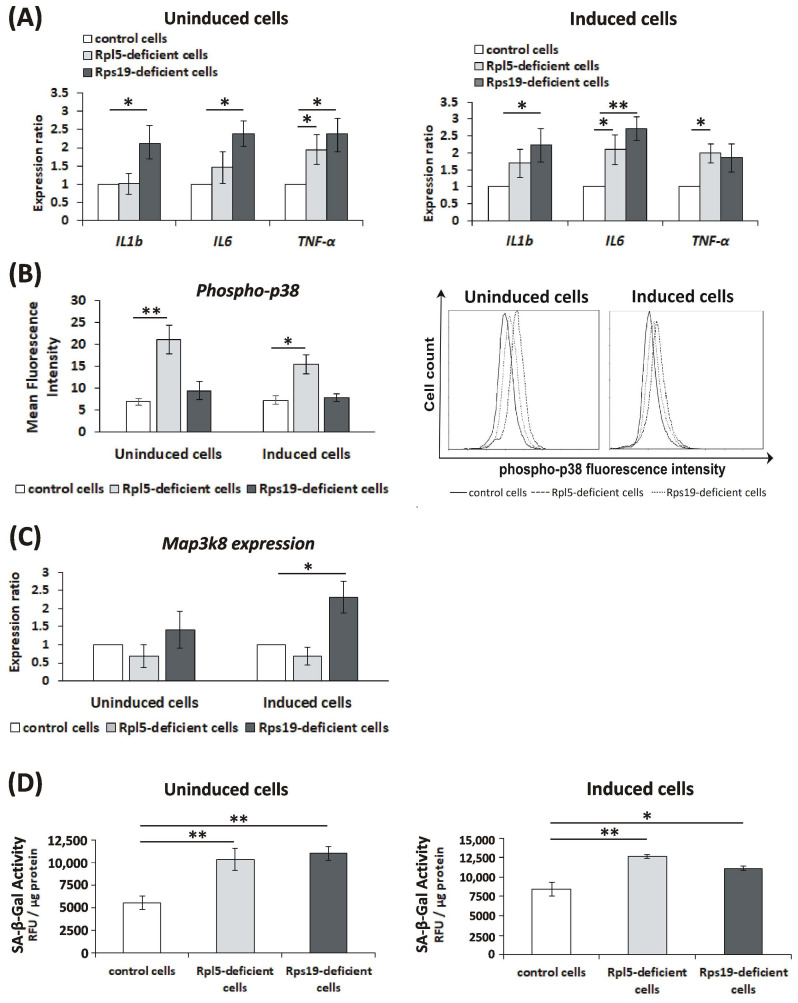
Proinflammatory cytokines expression, signaling pathways, and senescence in Rpl5- and Rps19-deficient murine erythroleukemia (MEL) cells. (**A**) Increased relative mRNA expression of interleukin 1b (*IL1b)*, *IL6*, and tumor necrosis factor-alpha (*TNF-α)* (normalized to beta-actin) in uninduced and induced Rpl5- and Rps19-deficient cells compared to controls. *p* values were calculated using the REST© 2020 software (Technical University Munich, Germany): * *p* < 0.05, ** *p* < 0.01; T bars designate standard error of the mean (SEM). (**B**) Significantly elevated phosphorylation of p38 kinase in uninduced and induced Rpl5-deficient cells compared to control cells; mean fluorescence intensity (MFI) of uninduced cells: 21.2 ± 3.2 for Rpl5-deficient cells vs. 6.9 ± 0.8 for control cells; MFI of induced cells: 15.5 ± 2.5 for Rpl5-deficient cells vs. 7.3 ± 0.9 for control cells. MFI for Rps19-deficient cells (uninduced: 9.5 ± 2.1 and induced: 7.8 ± 0.8) was comparable to controls. Values are given as mean ± standard deviation (SD). *p* values were calculated using the Student *t*-test; * *p* < 0.05, ** *p* < 0.01. (**C**) Significantly increased expression of *Map3k8* mRNA (normalized to beta-actin) in induced Rps19-deficient cells. *p* values were calculated using the REST© 2020 software (Technical University Munich, Germany): * *p* < 0.05; T bars designate SEM. (**D**) Significantly augmented SA-β-gal activity (relative fluorescence unit—RFU/μg protein) in uninduced and induced Rpl5-deficient (10,330 ± 1227 and 12,626 ± 221) and Rps19-deficient (10,983 ± 703 and 11,136 ± 307) cells compared to control cells (5566 ± 735 and 8435 ± 862). The absorbance was read at 360 nm (excitation)/ 465 nm (emission) on fluorescence reader (GENios, Tecan, Männedorf, Switzerland). Values are given as mean ± SD; the assay was repeated four times. The values were normalized to total protein levels; * *p* < 0.05, ** *p* < 0.01.

It is known that oxidative damage may be caused by exposure of cells to inflammatory cytokines [26]. In this context, upregulation of a proinflammatory cytokine tumor necrosis factor-alpha (TNF-α) was previously reported in RPS19-deficient hematopoietic progenitors and rps19-deficient zebrafish [27]. Indeed, upregulated expression of *TNF-α, interleukin 6* (*IL6*), and *IL1b* was detected by real-time PCR in Rpl5- and Rps19-deficient cells, both uninduced and induced, compared to control cells (Figure 4A). This was associated with significantly increased phosphorylation of p38 kinase (measured by flow cytometry) (Figure 4B) or induced expression of mitogen-activated protein kinase kinase kinase 8 (*Map3k8* kinase) mRNA (determined by real-time PCR) (Figure 4C) in Rpl5- and Rps19-deficient cells, respectively. Both of these molecules are known to play an essential role in the production of proinflammatory cytokines [28,29]. Proinflammatory cytokines may contribute to (as well as may result from) the induction of cellular senescence [30]. Significantly elevated senescence-associated β-galactosidase (SA-β-gal) activity was detected in both uninduced and induced Rpl5- and Rps19-deficient cells compared to control cells using the cellular senescence assay kit (Figure 4D).

**Figure 5:**
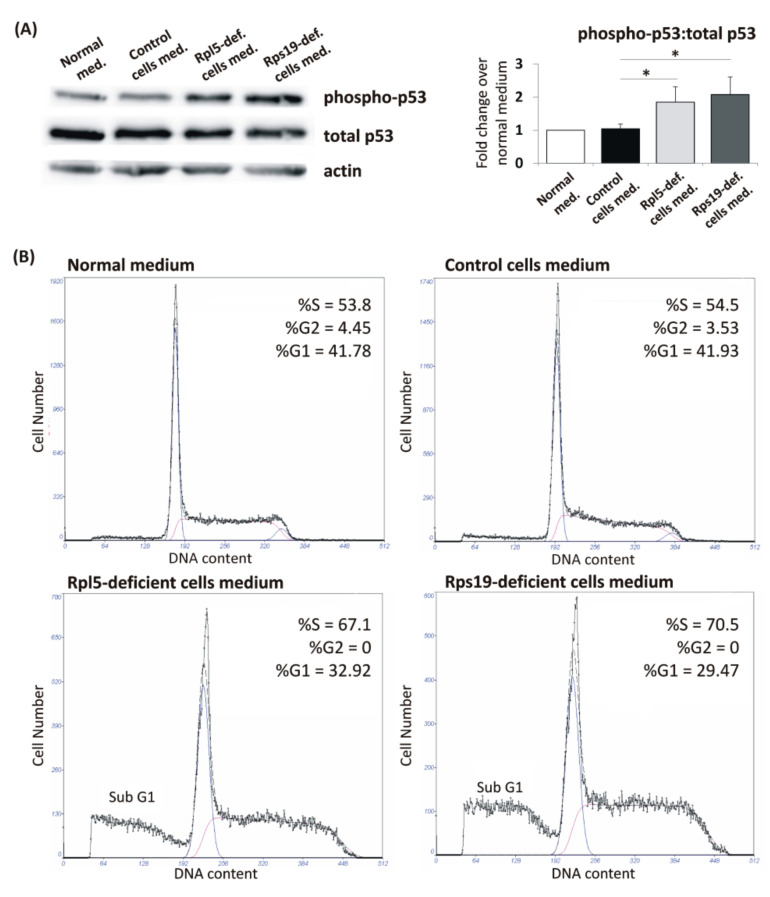
Effect of conditioned medium harvested from Rpl5- and Rps19-deficient cells on p53 activation and cell cycle of control cells. (**A**) Control cells cultivated in conditioned medium harvested from Rpl5- and Rps19-deficient cells showed activation of p53 (phospho-p53). In contrast, p53 phosphorylation in control cells cultivated in normal culture medium and those cultivated in conditioned medium harvested from control cells was comparable. A representative immunoblot is shown. Data in the bar graph showed a fold change in phospho-p53:total p53 ratio over cells cultured in normal cell culture medium and are expressed as means ± standard errors of the mean from three independent experiments; * *p* < 0.05 versus conditioned control cells medium. G:BOX-CHEMI-XX9 imaging system (Syngene, Cambridge, UK) was used for densitometry. (**B**) Control cells cultivated in conditioned medium harvested from Rpl5- and Rps19-deficient cells showed accumulation in the S phase and reduction in the G2/M phase of cell cycle. An increase in sub G1 fraction can also be noted. There was no difference between the cell cycle distribution of controls cells cultivated in normal culture medium and those cultivated in conditioned medium harvested from control cells. Similar data were obtained in three independent experiments.

To assess the involvement of cell-intrinsic production of cytokines and other secreted factors in the pathologies observed in Rpl5- and Rps19-deficient cells, we tested if a conditioned medium from mutant cells can induce cell-nonautonomous responses (bystander effects) in control cells [31]. Uninduced control cells were cultured in conditioned medium harvested from Rpl5- and Rps19-deficient cells as described in Figure S6; conditioned medium harvested from control cells served as a negative control. Immunoblot analysis of corresponding protein lysates showed activation of p53 (phospho-p53) in control cells cultured in conditioned medium harvested from Rpl5- and Rps19-deficient cells (Figure 5A). Simultaneously, propidium iodide (PI) staining revealed accumulation of control cells in the S-phase of cell cycle and an increased proportion of the sub G1 fraction (Figure 5B). These results demonstrate that cell-autonomous production of secreted factors/cytokines by Rpl5- or Rps19-deficient cells may inhibit cell cycle progression and activate p53 checkpoint and as such potentiate cell damage.

Because inflammatory cytokines appeared to be important contributors to DNA damage in Rp-deficient cells in our experiments, next, the effect of pomalidomide, a known TNF-α, IL1b and IL6 inhibitor [32], was tested. Addition of pomalidomide to the culture medium diminished 8-oxoG-positivity in Rpl5- and Rps19-deficient cells (Figure S7A,B) and concomitantly mitigated p53 activation in uninduced Rpl5- and Rps19-deficient cells (Figure S7C). The observed effect of pomalidomide on Rpl5- and Rps19-deficient cells can be attributed to the inhibition of *TNF-α*, *IL1b*, and *IL6* expression (Figure S8A) rather than changes in cell cycle progression [33] (Figure S8B).

### 2.6. Oxidative DNA Damage, Elevated Inflammatory Cytokines, and Activated DDR Signaling in DBA Patients

**Figure 6:**
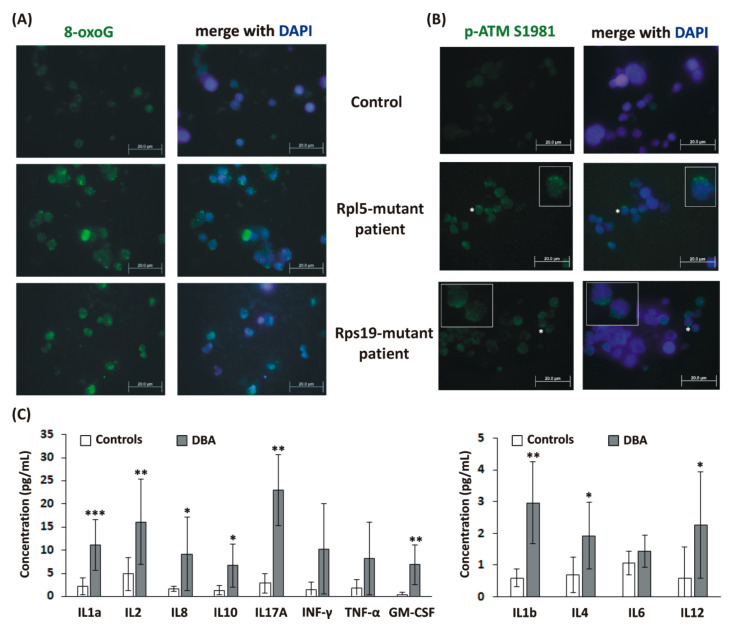
Oxidative DNA damage, p-ATM, and inflammatory cytokines in Diamond-Blackfan anemia (DBA) patients’ samples. (**A**) Increased numbers of 8-oxoguanine (8-oxoG)-positive (green color) RPL5-mutant erythroblasts (patient P2: 18.9%, patient P4: 5.7%) and RPS19-mutant erythroblasts (patient P7: 5.9%, patient P8: 6.9%) compared to control erythroblasts (*n* = 3, mean: 0.5 ± 0.2%). (**B**) Phosphorylated ATM at S1981 (p-ATM S1981, green color) in RPL5- and RPS19-mutant erythroblasts in comparison to control erythroblasts; representative images obtained for patient P1 and P7, and one control are shown. Cells nuclei were counterstained with 4′,6-diamidino-2-phenylindole dihydrochloride (DAPI) (blue color). The asterisks indicate cells shown in the insets. The slides were analyzed with an Olympus BX 51 fluorescence microscope (Olympus), original magnification 1000×. Digital images were acquired with an Olympus DP 50 camera driven by DP controller software (provided by Olympus, Tokyo, Japan). Images were cropped, assembled, and labeled using Adobe Photoshop software (Adobe Systems, San Jose, CA, USA). (**C**) Elevated cytokines in DBA patients’ serum (*n* = 11) compared to controls (*n* = 11). Values are given as mean ± standard deviation (SD). *p* values were calculated using the Student *t*-test; * *p* < 0.05, ** *p* < 0.01, and *** *p* < 0.001.

In order to validate the data obtained in DBA cellular models, erythroblast cell cultures derived from mononuclear cells (MNCs) isolated from selected RPL5- and RPS19-mutant patients (Table 1) were established as we previously described [34]. Subsequent ICC analyses revealed increased numbers of 8-oxoG-positive RPL5- and RPS19-mutant erythroblasts compared to control erythroblasts (Figure 6A). In addition, phosphorylated ATM at S1981 (p-ATM S1981), localized primarily in the cytoplasm, was observed in RPL5- and RPS19-mutant erythroblasts, but not in controls (Figure 6B).

Next, elevated levels of several cytokines were detected in the serum of DBA patients (*n* = 11). In particular, IL1a, IL1b, IL2, IL4, IL8, IL10, IL12, IL17a, and granulocyte-macrophage colony-stimulating factor (GM-CSF) were significantly increased compared to controls (C). Importantly, eight out of eleven analyzed patients were in disease remission. This confirms induction of inflammatory cytokine signature in DBA patients in vivo.

Finally, IHC analyses of DBA patients’ bone marrow samples (*n* = 9, Table 1) revealed elevated p53 positivity and apoptosis, which were not limited to erythroid cells (Figure S9). Moreover, sporadic 8-oxoG-positive bone marrow cells belonging to granulocyte cell lineage were detected in three patients (P2, P9, and P23) (Figure S10). In agreement, the p-ATM S1981 immunoreactivity detected in the cytoplasm of rare cells in patients P2 and P23 (Figure S11) indicated ATM phosphorylation in response to elevated ROS. However, in the RPS19-mutant patient P9, the number of p-ATM S1981 positive cells dramatically increased and the p-ATM S1981 signal became detectable not only in the cytoplasm but also in the cell nuclei, reflecting accumulation of DNA damage and activation of DNA damage signaling [35]. Importantly, patients P2 and P9 recently developed MDS. Altogether, this suggests that cells of different lineages (other than erythroid) may be vulnerable to DNA damage due to inherited RP-haploinsufficiency. Simultaneously, however, noncell autonomous (microenvironment-dependent) inflammatory stress may fuel oxidative damage of RP-deficient hematopoietic cells (as proposed by the experiment with conditioned medium shown in Figure 5).

## 3. Discussion

In this study, we used Rpl5- and Rps19-deficient cellular models and DBA patients’ samples to show that the ROS generation in Rp-mutant cells overpowers their antioxidant capacity, leading to oxidative DNA damage in erythroid precursors and altered characteristics of mature erythrocytes. In addition, our results imply inflammatory cytokines as mediators associated with oxidative stress in DBA cells. The above-mentioned features are shared to both ribosomal protein deficiencies with a certain degree of variation in the intensity of pathological phenotype.

Two aspects of DBA pathobiology have been evaluated in our study. The first one involved the assessment of consequences of oxidative stress on the properties of DBA RBCs. Mature erythrocytes are highly susceptible to oxidative damage and, therefore, possess several mechanisms, involving both nonenzymatic antioxidants and enzymatic antioxidants, to avoid excessive ROS formation [36]. It was documented that elevated ROS levels induce PS exposure to the outer surface of the erythrocyte membrane leading to enhanced clearance of erythrocytes by macrophages and reduced erythrocyte lifespan [6]. Excessive eryptosis has been described for multiple clinical conditions [8]. Here, we observed induced exposure of PS to the outer surface of the DBA erythrocyte membrane together with elevated ROS and despite the upregulation of antioxidant defense (Figure 1). This might indicate enhanced recognition of DBA erythrocytes by macrophages in vivo. As severe anemia is associated with tissue hypoxia, and ROS generation in hypoxia often exceeds the antioxidant buffering system of erythrocytes [37], profound hypoxemia may limit the antioxidant capacity of DBA erythrocytes. Moreover, studies on animal models and clinical evidence have indicated that erythrocytes are also sensitive to the presence of inflammatory cytokines, even at low levels of chronic inflammation [38]. In particular, IL8, one of the cytokines elevated in the serum of our DBA patients (Figure 6C), was shown to induce pathological changes to the erythrocyte membrane typical for eryptosis [38]. Based on these results, we propose that DBA erythrocytes have limited ROS buffering capacity which makes them more vulnerable to induced stress. Enhanced oxidative stress in DBA erythrocytes, for example during infections, may reinforce erythrophagocytosis and thus contribute to worsening of anemia. Supportive antioxidant treatment might therefore be beneficial in these conditions.

The second aspect addressed here was the extent of oxidative damage in developing DBA erythroblasts. An earlier study proposed the involvement of dysbalanced globin-heme synthesis in excessive ROS formation, predominantly in RPL5- and RPL11-deficient cells [3]. Using the CRISPR/Cas9-created Rpl5- and Rps19-deficient MEL cells, we observed insufficient capacity of both Rpl5- and Rps19-deficient cells to cope with higher ROS production (Figure 2 and Figure S4). This resulted in oxidative DNA damage, detected by increased 8-oxoG and γ-H2AX accumulation (Figure 2), and in the activation of ATR-Chk1 pathway (Figure 3A). This is consistent with previous reports showing downregulation of ROS scavengers in rpl11-mutant zebrafish and RPS19-deficient cells [39,40] and induction of γ-H2AX and activation of DDR in an rps19-deficient zebrafish model [5]. Nevertheless, the staining against p-ATM at S1981 revealed predominantly cytoplasmic p-ATM immunoreactivity in both Rpl5- and Rps19-deficient MEL cells (Figure 3B). Weak nuclear positivity, detected only in Rpl5-deficient cells, indicated low levels of DNA damage. Importantly, these data were confirmed in DBA patients’ erythroblasts derived in in vitro cultures where increased 8-oxoG positivity (Figure 6A) and cytoplasmic p-ATM immunoreactivity (Figure 6B) were detected. Thus, ATM in Rp-deficient cells exerts its actions mainly from the cytoplasm where it responds to the overproduction of ROS [20]. The cytoplasmic p-ATM was shown to regulate autophagy in order to maintain redox homeostasis [41]. Consistently, the induction of autophagy was previously observed in the cells with the knock-down of *RPS19* [4].

We also showed that in response to DNA damage, Rpl5- and Rps19-deficient cells activate DNA repair signaling molecules (Figure 3C). Nevertheless, the activation might be insufficient to completely prevent genomic instability. In agreement, leukemia-associated somatic RP-mutants induce oxidative stress and excessive DNA damage [42,43]. Increased risk of cancer, including both solid tumors and hematological malignancies (primarily MDS and acute myeloid leukemia), is reported in DBA [1]. Even though DBA primarily presents with the erythroid phenotype, the RP-haploinsufficiency is inherited to every cell in the body and the ongoing nucleolar and ribosomal stress may presensitize RP-haploinsufficient cells to DNA damage. Indeed, nucleolar stress can lead to cell cycle arrest and/or apoptosis in a p53-dependent manner [44,45]. Consistently, cells showing p53-positivity, apoptosis, 8-oxoG foci, activation of ATM kinase and belonging to the granulocyte lineage, were detected in the bone marrow of DBA patients from our cohort (Figures S9–S11). Importantly, three of the analyzed patients recently progressed to MDS.

**Figure 7:**
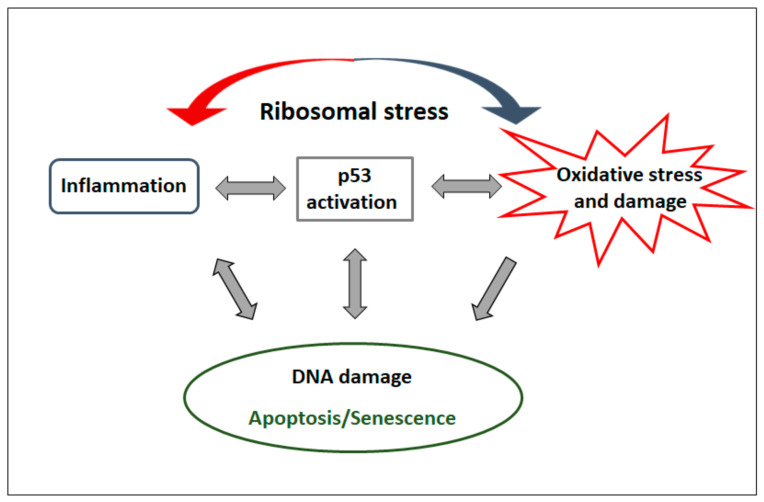
Interplay of deregulated pathways in Diamond-Blackfan anemia (DBA). Ribosomal protein (RP)-haploinsufficiency induces ribosomal stress leading to the activation of p53, induction of inflammatory signaling, and oxidative stress/damage. Multiple feedback loops between DNA damage response (DDR)/p53 activation and inflammatory cytokine production and between p53 activation and cell fate decisions (apoptosis/senescence) is expected. Similarly, a crosstalk between inflammatory cytokines and oxidative damage is presumed. Cells with activated DDR/p53 and exposed to inflammation and reactive oxygen species (ROS) may undergo senescence or apoptosis depending on the cellular context and DDR signaling thresholds. Damaged/senescent cells may further reinforce inflammatory cytokine production. The detailed hierarchical nature of cooperation between each deregulated pathway in DBA pathobiology needs to be defined.

Finally, our study underscored proinflammatory cytokines as important contributors to oxidative damage in Rpl5- and Rps19-deficient cells. The induction of proinflammatory cytokines in Rpl5- and Rps19-deficient cells in vitro (Figure 4A–C) and in the serum of DBA patients in vivo (Figure 6C) is consistent with previous studies reporting on a chronic subclinical inflammatory microenvironment in DBA bone marrow [46]. It is also in agreement with the upregulation of interferon (INF) signaling, inflammatory cytokines and mediators, and the complement system in rps19- and rpl11-deficient zebrafish [47]. This increased production of proinflammatory cytokines may either directly (via regulation of gene expression) or indirectly (via induction of cellular senescence) result from the activation of p53 in response to ribosomal stress (Figure 7) [48,49]. A positive feedback loop between p53 and proinflammatory cytokines can also be expected (Figure 7) [27,48,49]. Indeed, inhibition of TNF-α, IL1b, and IL6 in Rpl5- and Rps19-deficient cells upon pomalidomide treatment resulted in the reduction of oxidative DNA damage and inactivation of p53 (Figure S7). Elevated inflammatory cytokines may reinforce cellular senescence [30] observed in Rpl5- and Rps19-deficient cells (Figure 4D) and senescence may be potentiated by excessive oxidative stress (Figure 7) [50]. The induction of senescence is not necessarily conflicting with apoptosis of Rpl5- and Rps19-deficient cells (Figure S2B). Cells with activated DDR and exposed to inflammation and ROS may undergo senescence or apoptosis [51], depending on the cellular context and DNA damage signaling thresholds (Figure 7). Both these cellular phenotypes may, therefore, contribute to bone marrow failure in DBA patients in vivo. Altogether, our data indicate that anti-inflammatory treatment might relieve pathological DBA features associated with oxidative damage and activated p53.

Although a defective microenvironment is not considered to be the major cause of DBA [1], our data strongly support the hypothesis that RP-deficient erythroid cells contribute to and/or induce the production of proinflammatory cytokines, leading to the formation of an inflammatory bone marrow microenvironment. Accordingly, many cytokines, including IL6 and IL8, are among the hub genes reported in RPS19 mutant DBA [52]. The seeming discrepancy between induced *TNF-α* gene expression and signaling in DBA models [27,46] and in our Rp-deficient cells (Figure 4A), and rather normal levels of TNF-α in the serum of our DBA patients (Figure 6C), may reflect the fact that circulating cytokine levels not necessarily correlate with cellular cytokine production [53]. Indeed, most cytokines act in a local microenvironment where they have autocrine and paracrine functions. Increased levels of proinflammatory cytokines (e.g., TNF-α, IL1b, INF-γ) are known to suppress erythropoiesis [54,55,56]. Moreover, our experiment with conditioned medium harvested from Rpl5- and Rps19-deficient cell cultures (Figure 5) suggested that factors secreted by RP-deficient cells could induce/potentiate checkpoint signaling and cell death in a cell-nonautonomous fashion [57]. Our in vivo data showing oxidative damage and p53 activation in nonerythroid cells in the bone marrow from DBA patients (Figures S9–S11) further support this hypothesis. Consistently, analysis of a mouse model of Fanconi anemia, another inherited bone marrow failure syndrome with defective DNA repair, showed that TNF-α exposure creates an environment for clonal selection of somatically mutated preleukemic stem cells, thus leading to leukemogenesis [58]. The link between oxidative damage, inflammatory cytokines, and preleukemia risk in DBA deserves further comprehensive analyses.

In conclusion, our study extends the concept of a complex interplay of multiple mechanisms converging to DBA development and contributing to the high heterogeneity of clinical symptoms and response to therapy observed in DBA patients. We propose that defective ribosomal biogenesis is associated with the induction of inflammatory cytokine signature and oxidative damage. Further research is needed to conclusively elucidate the hierarchy of all deregulated pathways in DBA. Nevertheless, the obtained results indicate that therapeutic interventions targeting elevated ROS and/or inflammatory cytokines could alleviate the DBA phenotype in vivo.

## 4. Materials and Methods

### 4.1. Patient Samples

DBA patients (*n* = 24) from a recently updated Czech and Slovak DBA registry [10] were included in the study. The patients’ basic characteristics can be found in Table 1. The control group consisted of age- and sex-matched healthy individuals. All patients´ and controls´ samples were obtained with an informed consent. The study was conducted in accordance with the Declaration of Helsinki and approved by the Ethics Committee of University Hospital Olomouc on 17 June 2015 (Project identification code 16-32105A).

#### 4.1.1. Determination of ROS

Erythrocytes from DBA patients and healthy controls were incubated with 0.4 mM DCF-DA (Sigma-Aldrich, Darmstadt, Germany) for 15 min at 37 °C according to Amer et al. [59] DCF-dependent intensity of fluorescence was measured by FACS Calibur (BD Bioscience, Franklin Lakes, NJ, USA).

#### 4.1.2. Erythrocyte Annexin V Binding

Annexin V/FITC kit was purchased from BD Biosciences (Franklin Lakes, NJ, USA) and the assay performed as previously described [11]. Fluorescence intensity was measured by FACS Calibur.

#### 4.1.3. Glutathione Measurements and Erythrocyte Enzyme Activity

GSH was determined using a quantification kit for oxidized and reduced glutathione (Sigma-Aldrich, Darmstadt, Germany) according to manufacturer´s instructions. Activity of enzymes involved in the pentose phosphate pathway and oxidative defense: G6PD, GLD, and GPx was determined according to the methods recommended by the International Committee for Standardization in Haematology [60], as we previously described [61,62]. Leukocyte- and platelet-free erythrocyte lysates were used, and the absorbance was measured by spectrophotometer (Infinite 200 Nanoquant; Tecan, Männedorf, Switzerland). All chemicals and purified enzymes were purchased from Sigma-Aldrich (Darmstadt, Germany).

#### 4.1.4. Erythroblast Cell Culture and ICC Analyses

MNCs were isolated from the whole peripheral blood using density centrifugation and cultivated according to previously published protocols [63,64], with minor changes. For the first seven days, 1 × 10^6^/mL MNCs were cultured in StemPro™-34 SFM medium (ThermoFisher Scientific, Waltham, MA, USA) containing L-glutamine (2 mM, ThermoFisher Scientific, Waltham, MA, USA), 1X cytokine cocktail StemSpan™ CC110 (StemCell Technologies, Vancouver, BC, Canada), recombinant human erythropoietin (EPO, 2 U/mL; StemCell Technologies, Vancouver, BC, Canada), and the synthetic glucocorticoid dexamethasone (Dex, 1 µM; Sigma-Aldrich, Darmstadt, Germany). To induce erythroid differentiation, proliferating erythroblasts were cultured in StemPro™-34 SFM medium supplemented with l-glutamine (2 mM), human stem cell factor (50 ng/mL, StemCell Technologies, Vancouver, BC, Canada), human insulin like growth factor-1 (50 ng/mL, StemCell Technologies, Vancouver, BC, Canada), EPO (10 U/mL), and holo-transferrin (1 mg/mL, Sigma-Aldrich, Darmstadt, Germany). The cultures were maintained at 37 °C in 5% CO_2_/95% air atmosphere with a medium changed every two days.

Differentiated cells (day 14) were cytospined on glass slides and fixed with 3% paraformaldehyde (PHA) and methanol solution. After permeabilization (0.1% Tween in PBS for 10 min), the slides were incubated with primary antibodies: 8-oxoG (clone 483.15, Santa Cruz Biotechnology, Dallas, TX, USA) or ATM pS1981 (clone 7C10D8, Rockland Immunochemicals, Pottstown, PA, USA) for 1 h. After washing, Alexa Fluor^®^488-conjugated secondary antibodies were used (both from ThermoFisher Scientific, Waltham, MA, USA). Cells nuclei were stained with 0.001% DAPI (Sigma-Aldrich, Darmstadt, Germany); cells were visualized using fluorescence microscopy.

#### 4.1.5. Determination of Inflammatory Cytokines Levels

Human Inflammatory Cytokines Multi-Analyte ELISArray™ Kit was used according to manufacturer’s instructions (Qiagen, Venlo, The Netherlands).

#### 4.1.6. Immunohistochemistry on Bone Marrow Samples

IHC staining was performed using formalin-fixed and paraffin-embedded bone marrow biopsy samples as we previously described [15,65] with the use of following antibodies: p53 (clone 7F5, Cell Signaling Technologies, Danvers, MA, USA), 8-oxoG (clone 2Q2311, Abcam, Cambridge, UK), and ATM pS1981 (clone 7C10D8, Rockland Immunochemicals, Pottstown, PA, USA) and an EnVision+ Dual Link Detection System (HRP and DAB+ as a visualization chromogen; both DAKO/Agilent, Santa Clara, CA, USA). The alkaline phosphatase (AP) in situ cell death detection kit (Roche Applied Science, Mannheim, Germany) was used according to the manufacturer’s instructions as we previously described [15]. The slides were analyzed by light microscopy.

### 4.2. Cell Lines

Rpl5- and Rps19-deficient MEL were prepared by CRISPR/Cas9 technology. Detailed information on cell transfection and protocol for genotyping of individual clones is given in Supplementary Materials. The cells were maintained in DMEM medium containing 10% fetal bovine serum (FBS; ThermoFisher Scientific, Waltham, MA, USA). Erythroid differentiation was induced with 1.8% DMSO for 96 h [12]. In selected experiments, pomalidomide (10 µM, Sigma-Aldrich, Darmstadt, Germany) was added to the culture medium [66].

#### 4.2.1. Proliferation Assay and Apoptosis

Rpl5- and Rps19-deficient MEL clones were plated on a 96-well plate. The thiazolyl blue tetrazolium bromide (MTT) proliferation assay was performed according to manufacturer’s instructions (Sigma-Aldrich, Darmstadt, Germany), as we previously described [67]. The fluorescein in situ cell death detection kit (Roche Applied Science, Mannheim, Germany) was used for apoptosis detection on cytospined slides of MEL cells as we previously described [68]; for more details see Supplementary Materials.

#### 4.2.2. Immunoblotting

MEL cells were lysed on ice in Radio-Immunoprecipitation Assay (RIPA) lysis buffer (ThermoFisher Scientific, Waltham, MA, USA) with 100 μM Na orthovanadate, 100 μM (phenylmethylsulfonyl fluoride) PMSF, and a cocktail of protease inhibitors (all from Sigma-Aldrich, Darmstadt, Germany). The following primary antibodies were used for immunoblotting: RPS19 (Abcam, Cambridge, UK), RPL5 (Abcam, Cambridge, UK), p53 (Cell Signaling Technologies, Danvers, MA, USA), phospho-p53 (Ser15, Cell Signaling Technologies, Danvers, MA, USA), Chk1 (clone G-4, Santa Cruz Biotechnology, Dallas, TX, USA), phospho-Chk1 (Ser345, clone 133D3, Cell Signaling Technologies, Danvers, MA, USA), tubulin (Cell Signaling Technologies, Danvers, MA, USA), and actin (Sigma-Aldrich, Darmstadt, Germany). The Western blots were analyzed by chemiluminescent detection method using SuperSignal™ West Dura Extended Duration Substrate (ThermoFisher Scientific, Waltham, MA, USA). The bands were detected by traditional x-ray film system or G:BOX-CHEMI-XX9 imaging system (Syngene, Cambridge, UK). ImageJ was used for densitometry of protein expression evaluated by traditional x-ray film system [69].

#### 4.2.3. Real-Time PCR Assay

RNA was isolated using TRI Reagent (Sigma-Aldrich, Darmstadt, Germany) and reverse-transcribed using SuperScript^®^ VILO™ cDNA Synthesis Kit (ThermoFisher Scientific, Waltham, MA, USA) or Transcriptor First Strand cDNA Synthesis Kit (Roche Applied Science, Mannheim, Germany) according to manufacturers’ instructions. Real-time polymerase chain reaction was performed in triplicates on a LightCycler 480 (Roche Applied Science, Mannheim, Germany) using TaqMan^®^ Gene Expression Master Mix (ThermoFisher Scientific, Waltham, MA, USA) or LightCycler^®^ 480 Probes Master Mix. For real-time PCR using the UPL probes (Roche Applied Science, Mannheim, Germany) cDNA was treated with Turbo DNA-free kit (ThermoFisher Scientific, Waltham, MA, USA). The list of TaqMan^®^ Gene Expression probes (ThermoFisher Scientific, Waltham, MA, USA) and UPL probes and primers can be found in Supplementary Material and Methods. The data were normalized to the expression of beta-actin and to mRNA levels of control cells. The statistical significance of relative expression changes of target mRNA levels was analyzed using REST© 2020 software (Technical University Munich, Germany) [70].

#### 4.2.4. Flow Cytometry Analysis of γ-H2AX and Phosphorylated p38

MEL cells were fixed with 3% PHA and permeabilized with ice-cold 70% ethanol. After the washing and blocking step (with 0.5% BSA/PBS), the cells were incubated with Alexa Fluor^®^488-conjugated antibody against phospho-histone H2AX (Ser139; Cell Signaling Technologies, Danvers, MA, USA) or primary antiphospho-p38 antibody (Cell Signaling Technologies, Danvers, MA, USA) for 1 h. For phospho-p38 detection, FITC-conjugated secondary antibody (BD Biosciences, Franklin Lakes, NJ, USA) was used for another hour. The intensity of fluorescence was measured by FACS Calibur. To induce γ-H2AX, control MEL cells were irradiated with γ-rays (4 Gy, 30 min) before staining.

#### 4.2.5. ROS Measurement

MEL cells were stained with 0.5 μM CellROX^®^ Green reagent (ThermoFisher Scientific, Waltham, MA, USA) for 45 min at 37 °C in the dark, washed with PBS, and fixed with 4% formaldehyde (ThermoFisher Scientific, Waltham, MA, USA) in PBS. The intensity of fluorescence was measured by FACS Calibur.

#### 4.2.6. ICC of 8-OxoG and ATM pS1981

ICC on cytospin slides of MEL cells was performed as described for differentiated erythroblasts (Section 4.1.4).

#### 4.2.7. Cellular Senescence Activity Assay

MEL cells were lysed in ice-cold lysis buffer (Enzo Life Sciences, Farmingdale, NY, USA) containing protease inhibitors for 15 min on ice. The measurement of SA-β-gal activity was performed according to manufacturer’s instructions (ENZ-kit129, Enzo Life Sciences Farmingdale, NY, USA) using a fluorescence reader (GENios, Tecan, Männedorf, Switzerland).

#### 4.2.8. Cell Cycle Analysis

The cells were harvested, fixed (ice-cold 70% ethanol), permeabilized (1% BSA/0.5% Tween-20), and stained with PI (Sigma-Aldrich, Darmstadt, Germany) for 30 min. Cell cycle was measured by flow cytometry (FACS Calibur) as previously described [67] and the cell cycle distribution was analyzed by MultiCycle AV software (Phoenix Flow System, San Diego, CA, USA).

### 4.3. Statistical Analyses

Student’s *t*-test was used to determine the statistical significance of the results. *p* values less than 0.05 were considered statistically significant. Statistical analyses were conducted using Origin 6.1 software (OriginLab Corporation, Northampton, MA, USA). Enzyme activity graphs were created, and the corresponding *p* values calculated using GraphPad Prism 8 Software (GraphPad Software Inc., La Jolla, CA, USA).

## Figures and Tables

**Table 1:** DBA patients included in the study.

Pct	Sex	Age	Gene Mutation	Treatment	ROS	Annexin V	ELISA Inflamm. Cytokines	GSH Measurements	RBC Enzyme Activities	In Vitro Cultivation	IHC
P1	F	*54*	*RPL5*	R	+	+	+	+	NA	+	NA
P2 *	M	*32*	*RPL5*	R	+	+	+	+	+	+	+
P3	F	*42*	*RPL5*	R	NA	+	+	+	+	NA	NA
P4 *	M	*33*	*RPL5*	R	+	+	+	+	+	+	+
P5	M	*20*	*RPS19*	S	+	+	+	NA	NA	NA	NA
P6	F	*19*	*RPS19*	S	+	+	+	NA	NA	NA	NA
P7	M	*40*	*RPS19*	R	+	+	+	+	+	+	NA
P8	M	*46*	*RPS19*	S, Leu	+	NA	+	+	+	+	NA
P9 *	M	*28*	*RPS19*	R	+	+	NA	+	+	NA	+
P10	F	*46*	*RPL11*	R	+	NA	+	NA	NA	NA	NA
P11	M	*8*	*RPL11*	S	+	+	NA	NA	NA	NA	NA
P12	F	*36*	*RPL11*	R	+	NA	NA	NA	NA	NA	NA
P13	F	*54*	*RPL11*	S	+	NA	NA	NA	NA	NA	NA
P14	F	*27*	*RPS7*	R	+	+	+	+	+	NA	NA
P15	F	*41*	*RPS7*	R	+	+	NA	+	+	NA	NA
P16	F	*54*	*RPS7*	R	+	+	NA	+	+	NA	NA
P17	F	*33*	*delRPL35a*	S	+	+	NA	NA	NA	NA	NA
P18	M	*34*	*No mut*	S	+	+	NA	+	+	NA	+
P19	M	*10*	*No mut*	R	+	+	+	NA	NA	NA	NA
P20	F	*30*	*RPS19*	T, DRX	NA	NA	NA	NA	NA	NA	+
P21	F	*34*	*RPS19*	T, DRX	NA	NA	NA	NA	NA	NA	+
P22	M	*26*	*RPS26*	T, DRX	NA	NA	NA	NA	NA	NA	+
P23	M	*21*	*RPS26*	T, DRX	NA	NA	NA	NA	NA	NA	+
P24 ^§^	F	*31* ^§^	*RPL11*	T, DRX	NA	NA	NA	NA	NA	NA	+

Pct—patient, ROS—reactive oxygen species, GSH—reduced glutathione, RBCs—red blood cells, IHC—immunohistochemistry, R—remission, S—steroids, Leu—leucine, T—transfusions, DRX—deferasirox, No mut—no mutation identified, NA—not analyzed, * P2, P4, and P9 recently developed myelodysplastic syndrome (MDS) with bicytopenia in peripheral blood and two or three-lineage dysplasia in the bone marrow with less than 5% of blasts. RPL5-mutant patients (P2 and P4) had no typical MDS changes by flow cytometry, cytogenetics or sequencing; an *ASXL1* mutation was detected in RPS19-mutant patient (P9) [10]. The material from these patients (including bone marrow biopsy) was collected before MDS development. ^§^ P24 deceased of triple-negative breast cancer in 2019.

## Supplementary Materials

The following are available online at https://www.mdpi.com/1422-0067/21/24/9652/s1. Figure S1: Creation of Rpl5- and Rps19-deficient MEL cells; Figure S2: Characterization of Rpl5- and Rps19-deficient MEL cells; Figure S3: Relative expression of *Gata1*; Figure S4: Activities of ROS scavenging enzymes in Rpl5- and Rps19-deficient cells; Figure S5: Positive controls for 8-oxoG staining; Figure S6: Effect of conditioned medium harvested from Rpl5- and Rps19-deficient cells on p53 activation and cell cycle of control cells; Figure S7: Immunocytochemistry staining for 8-oxoG and immunoblot analysis for phospho-p53 protein in Rpl5- and Rps19-deficient cells; the effect of pomalidomide; Figure S8: Effect of pomalidomide on the expression of inflammatory cytokines and cell cycle progression in uninduced Rpl5- and Rps19-deficient cells; Figure S9: Immunohistochemical staining of bone marrow specimens; Figure S10: Immunohistochemistry for 8-oxoG in the bone marrow specimens; Figure S11. Immunohistochemistry for pATM in the bone marrow specimens.
