# Supplementary material for: Oxidative DNA Damage, Inflammatory Signature, and Altered Erythrocytes Properties in Diamond-Blackfan Anemia
Source: Int J Mol Sci. 2020 Dec 17;21(24):9652. doi: 10.3390/ijms21249652 (PMC7768356; doi:10.3390/ijms21249652)
Supplement: Supplementary file 1 [file ijms-21-09652-s001.pdf]

Kapralova et al., Oxidative DNA damage, inflammatory signature, and altered erythrocytes properties in Diamond-Blackfan anemia

Supplementary Material and Methods and Figures

Supplementary Material and Methods

Bacteria and plasmids

The LentiCRISPR single guide RNA plasmid (#49535, Addgene, Watertown, MA, USA) was digested by *BsmB I* enzyme, purified from agarose gel (Roche, High Pure PCR Product Purification Kit) and ligated with phosphorylated and annealed oligo pairs for single guide RNA (target sequence for *Rpl5* exon 3: 5'-AATATAGGATGATAGTTCGT-3', for *Rps19* exon 4: 5'-GGCCGCAAGCTGACGCCTCA-3'). The selected guide RNA recognition sequences (gRNAs) with corresponding protospacer adjacent motifs (PAM sequences) were also cloned into the pAR-TurboRFP plasmid (#60021, Addgene), which enables functional test of activity and selectivity of gRNA. A CRISPR/Cas9-mediated cut causes expression of green fluorescent protein (GFP) in LentiCRISPR single guide RNA plasmid and red fluorescent protein (RFP) in pAR-TurboRFP plasmid providing markers for fluorescence activated cell sorting (FACS).

Cell lines

MEL cells, transfection, and selection of stable transfectants

MEL cells [1] were cultured in DMEM medium containing 10% fetal bovine serum (FBS) (both from ThermoFisher Scientific, Waltham, MA, USA). A total number of 3 x 10^6 cells in 10 mL of Opti-MEM medium (ThermoFisher Scientific) were collected for each transfection using Lipofectamine 3000 Transfection Reagent (ThermoFisher Scientific). The co-transfection of LentiCRISPR/Cas9 plasmid (10 µg) and pAR-TurboRFP reporter plasmid (10 µg) was performed according to manufacturer's instructions. After 24 hours, the individual cells were sorted according the expression of similar levels of GFP and RFP fluorescence by BD Influx high speed cell sorter (BD Biosciences). The particular CRISPR/Cas9-created clones were heterozygous for different nucleotide size deletions in *Rpl5* or *Rps19* genes resulting in decreased levels of corresponding proteins to half when compared to control cells (transfected with reporter plasmid) thus mimicking the RP haploinsufficiency in DBA patients (Supplemental Figure S1). Control cells were produced by the same principle with reporter plasmid transfection and sorted according the expression of similar levels of GFP. All cells were prepared in parallel.

Genotyping of MEL clones

Genotyping of Rpl5- and Rps19-deficient MEL clones was based on detection of nucleotide deletion in *Rpl5* and *Rps19* genes using PCR reaction followed by gel electrophoresis. Genomic DNA was isolated by Gentra Puregene Cell Kit (Qiagen). The PCR reaction was performed using HotStarTaq Master Mix Kit (Qiagen) as recommended by manufacturer. For detection of *Rpl5* deletion: initial denaturation 95°C/15 minutes, then 33

cycles of: 95°C/10 seconds, 52°C/30 seconds, 72°C/30 seconds, with final elongation at 72°C/5 minutes; for *Rps19* deletion: initial denaturation 95°C/15 minutes, then 35 cycles of: 95°C/30 seconds, 58°C/30 seconds, 72°C/30 seconds, final elongation at 72°C/5 minutes. Subsequent gel electrophoresis was performed using the high resolution sieving agarose SFR (VWR, Radnor, PA, USA). G:BOX-CHEMI-XX9 Gel Documentation System (Syngene) was used for gel imaging. Sequences of primers used for amplification of target part of *Rpl5* and *Rps19* genes are listed below.

Rpl5 (F) 5´- TGCTCGAAAACGATTGGTGA - 3´
Rpl5 (R) 5´- AGGACATAACTCACCTGGCA - 3´
Rps19 (F) 5´- GCTGTACTCATCCAGGGTTTG - 3´
Rps19 (R) 5´- CTCTCACCTGTCCAGCGATC - 3´

Real-time PCR assay

For real-time PCR using the UPL probes (Roche Applied Science) cDNA was treated with Turbo DNA-free kit (ThermoFisher Scientific).

The list of TaqMan® Gene Expression used for real-time PCR:

Rpl5 (#Mm00847026), Rps19 (#Mm01611010), Gata1 (#Mm01352636), Sod1 (#Mm01344233_g1), Sod2 (#Mm01313000_m1), Cat (#Mm01340251_m1), Recql4 (Mm00453659_m1), Ogg1 (#Mm00501781_m1), Prkdc (#Mm01342967_m1), and beta-actin (#4352933E).

The list of UPL probes and primers used for real-time PCR:

Map3k8 - probe n. 4 (cat. no. 04685016001)
F 5´- GAC TCT GCC CTC TTT GAA CG - 3´
R 5´- GTG CTG CCT GTG CAT GAT - 3´

Tnf-alpha - probe n. 49 (cat. no. 04688104001)
F 5´- TGC CTA TGT CTC AGC CTC TTC - 3´
R 5´- GAG GCC ATT TGG GAA CTT CT - 3´

Beta-actin - probe n. 56 (cat. no. 04688538001)
F 5´- AAG GCC AAC CGT GAA AAG AT - 3´
R 5´- GTG GTA CGA CCA GAG GCA TAC -3´

Il1b2 - probe n. 78 (cat. no. 04689011001)
F 5´- TGT AAT GAA AGA CGG CAC ACC - 3´
R 5´- TCT TCT TTG GGT ATT GCT TGG - 3´

Il6 - probe n. 6 (cat. no. 04685032001)
F 5´- GAT GGA TGC TAC CAA ACT GGA T - 3´
R 5´- CCA GGT AGC TAT GGT ACT CCA GA - 3´

Evaluation of apoptosis - TUNEL assay

The Fluorescein *In Situ* Cell Death Detection Kit (Roche Applied Science) was used to perform immunocytochemistry for apoptosis detection on cytospined slides of uninduced

MEL cells as we previously described [2]. Apoptotic cells were identified as being Terminal deoxynucleotidyl transferase dUTP nick end labeling (TUNEL)-positive (TUNEL+) and DAPI+. DAPI (0.001%; Sigma-Aldrich) was used to counterstained cell nuclei. Slides were analyzed using immunofluorescence microscopy.

Enzyme activity assay

Activity of enzymes involved in oxidative defense, particularly glucose-6-phosphate dehydrogenase (G6PD), gluconolactone dehydrogenase (GLD), and glutathione peroxidase (GPx), was determined according to the methods recommended by the International Committee for Standardization in Haematology [3], as we previously described [4,5]. Cell lysates from uninduced and induced MEL cells were used and the absorbance was measured in spectrophotometer (Infinite 200 Nanoquant; Tecan, Männedorf, Switzerland). Specific enzyme activity was calculated using the Lambert-Beer law. All chemicals and purified enzymes were purchased from Sigma-Aldrich.

References:

1. Antoniou, M. Induction of Erythroid-Specific Expression in Murine Erythroleukemia (MEL) Cell Lines. *Methods Mol Biol.* **1991**, *7*, 421-434.

2. Horvathova, M.; Kapralova, K.; Zidova, Z.; Dolezal, D.; Pospisilova, D.; Divoky, V. Erythropoietin-driven signaling ameliorates the survival defect of DMT1-mutant erythroid progenitors and erythroblasts. *Haematologica* **2012**, *97*, 1480-1488.

3. Beutler, E.; Blume, K.G.; Kaplan, J.C.; Löhr, G.W.; Ramot, B.; Valentine, W.N. International Committee for Standardization in Haematology: recommended methods for red-cell enzyme analysis. *Br J Haematol.* **1977**, *35*, 331-340.

4. Mojzikova, R.; Dolezel, P.; Pavlicek, J.; Mlejnek, P.; Pospisilova, D.; Divoky, V. Partial glutathione reductase deficiency as a cause of diverse clinical manifestations in a family with unstable hemoglobin (Hemoglobin Haná, β63(E7) His-Asn). *Blood Cells Mol Dis.* **2010**, *45*, 219-222.

5. Mojzikova, R.; Koralkova, P.; Holub, D.; Zidova, Z.; Pospisilova, D.; Cermak, J.; Striezencova Laluhova, Z.; Indrak, K.; Sukova, M.; Partschova, M.; et al. Iron status in patients with pyruvate kinase deficiency: neonatal hyperferritinaemia associated with a novel frameshift deletion in the PKLR gene (p.Arg518fs), and low hepcidin to ferritin ratios. *Br J Haematol.* **2014**, *165*, 556-563.

6. Pfaffl, M.W.; Horgan, G.W.; Dempfle, L. Relative expression software tool (REST) for group-wise comparison and statistical analysis of relative expression results in real-time PCR. *Nucleic Acids Res.* **2002**, *30*, e36.

7. Schneider, C.A.; Rasband, W.S.; Eliceiri, K.W. NIH Image to ImageJ: 25 years of image analysis. *Nature methods* **2012**, *9*, 671-675.

8. Pospisilova, D.; Holub, D.; Zidova, Z.; Sulovska, L.; Houda, J.; Mihal, V.; Hadacova, I.; Radova, L.; Dzubak, P.; Hajduch, M.; et al. Hepcidin levels in Diamond-Blackfan anemia reflect erythropoietic activity and transfusion dependency. *Haematologica* **2014**, *99*, e118-121.

Figure S1:

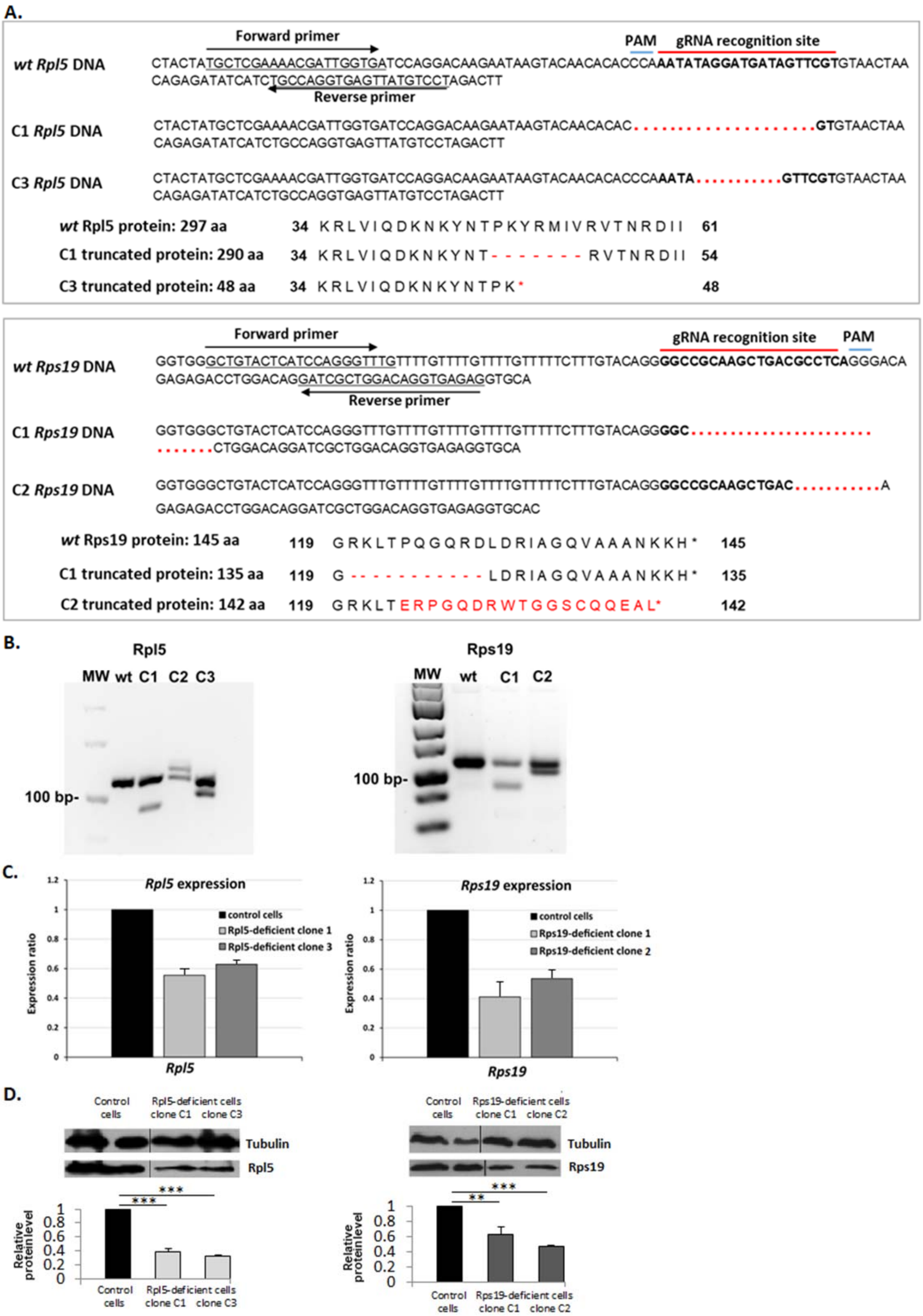
A.
Forward primer
PAM
gRNA recognition site
wt Rpl5 DNA
CTACTATGCTCGAAAACGATTGGTGATCCAGGACAAGAATAAGTACAACACACCCAAATATAGGATGATAGTTCGTGTAACTAA
CAGAGATATCATCTGCCAGGTGAGTTATGTCCTAGACTT
Reverse primer
C1 Rpl5 DNA
CTACTATGCTCGAAAACGATTGGTGATCCAGGACAAGAATAAGTACAACACAC..................GTGTAACTAA
CAGAGATATCATCTGCCAGGTGAGTTATGTCCTAGACTT
C3 Rpl5 DNA
CTACTATGCTCGAAAACGATTGGTGATCCAGGACAAGAATAAGTACAACACACCCAAATA..........GTTCGTGTAACTAA
CAGAGATATCATCTGCCAGGTGAGTTATGTCCTAGACTT
wt Rpl5 protein: 297 aa
34 KRLVIQDKNKYNTPKYRMIVRVTNRDII 61
C1 truncated protein: 290 aa
34 KRLVIQDKNKYNT-------RVTNRDII 54
C3 truncated protein: 48 aa
34 KRLVIQDKNKYNTPK* 48
Forward primer
gRNA recognition site
PAM
wt Rps19 DNA
GGTGGGCTGTACTCATCCAGGGTTTGTTTTGTTTTGTTTTGTTTTTCTTTGTACAGGGGCCGCAAGCTGACGCCTCAGGGACA
GAGAGACCTGGACAGGATCGCTGGACAGGTGAGAGGTGCA
Reverse primer
C1 Rps19 DNA
GGTGGGCTGTACTCATCCAGGGTTTGTTTTGTTTTGTTTTGTTTTTCTTTGTACAGGGGC......................
.......CTGGACAGGATCGCTGGACAGGTGAGAGGTGCA
C2 Rps19 DNA
GGTGGGCTGTACTCATCCAGGGTTTGTTTTGTTTTGTTTTGTTTTTCTTTGTACAGGGGCCGCAAGCTGAC...........A
GAGAGACCTGGACAGGATCGCTGGACAGGTGAGAGGTGCAC
wt Rps19 protein: 145 aa
119 GRKLTPQGQRDLDRIAGQVAAANKKH* 145
C1 truncated protein: 135 aa
119 G-----------LDRIAGQVAAANKKH* 135
C2 truncated protein: 142 aa
119 GRKLTERPGQDRWTGGSCQQEAL* 142
B.
Rpl5
MW wt C1 C2 C3
100 bp-
Rps19
MW wt C1 C2
100 bp-
C.
Rpl5 expression
control cells
Rpl5-deficient clone 1
Rpl5-deficient clone 3
Expression ratio
Rpl5
Rps19 expression
control cells
Rps19-deficient clone 1
Rps19-deficient clone 2
Expression ratio
Rps19
D.
Control cells
Rpl5-deficient cells clone C1 clone C3
Tubulin
Rpl5
Relative protein level
Control cells
Rpl5-deficient cells clone C1 clone C3
Control cells
Rps19-deficient cells clone C1 clone C2
Tubulin
Rps19
Relative protein level
Control cells
Rps19-deficient cells clone C1 clone C2

Figure S1: Creation of Rpl5- and Rps19-deficient MEL cells

A. Generation of Rpl5 and Rps19 mutants. Corresponding DNA and protein sequences are aligned to show the CRISPR/Cas9-targeted region of *Rpl5* (top) and *Rps19* (bottom) gene, respectively. The gRNAs are in bold and marked by a red color. The PAM sequences are overlined (blue color) and the genotyping primers are underlined. A CRISPR/Cas9-mediated cut and subsequent repair of genomic DNA by non-homologous end joining generated different lengths of nucleotide deletions in the coding sequence of the *Rpl5* and *Rps19* genes, respectively. These DNA alterations resulted in a premature stop of translation and in a truncated protein. Amino acid residues translated upon the frameshift are shown in red; asterisks indicate premature termini of the protein and dashes indicate missing amino acids. Numbers indicate the positions in the translated portion of protein. **B. Genotyping of Rpl5- and Rps19-deficient clones.** PCR analysis and subsequent gel electrophoresis revealed deletions and insertion in *Rpl5* and *Rps19* genes. **C. Rpl5 and Rps19 mRNA expression in Rpl5- and Rps19-deficient clones.** Real-time PCR analysis showed reduction of *Rpl5* and *Rps19* transcripts (normalized to beta-actin) in Rpl5- and Rps19-deficient clones (C1, C3 and C1, C2, respectively) in comparison with control cells as calculated by the REST© 2009 software [6]. **D. Immunoblot analysis of Rpl5 and Rps19 proteins.** Decreased levels of Rpl5 and Rps19 proteins were detected in Rpl5- and Rps19-deficient cells (clones C1, C3 and C1, C2, respectively) in comparison with control cells. A representative immunoblot is shown. Tubulin served as loading control for normalization. Data in the bar graph are expressed as means ± standard errors of the mean from 3 independent experiments; **$p < 0.01$ and ***$p < 0.01$ versus control cells. ImageJ was used for densitometry [7].

Figure S2:

A.

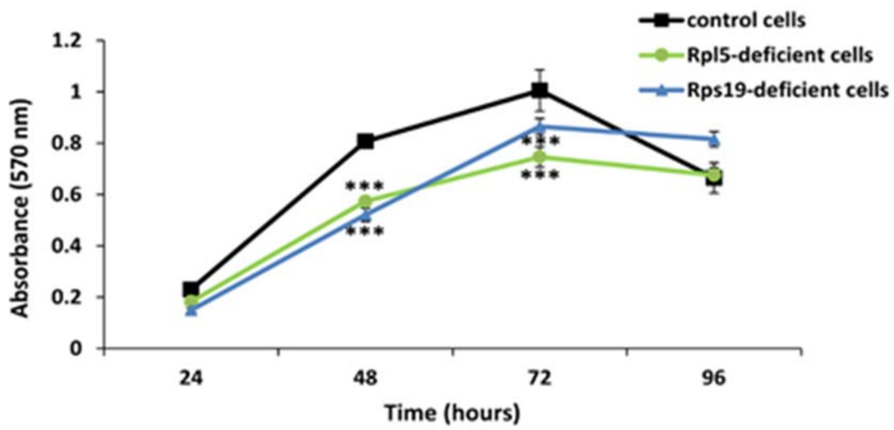

B.

% of TUNEL⁺ and DAPI⁺ MEL cells	
**Rpl5-deficient cells**	4.81 ± 1.73 %
**Rps19-deficient cells**	1.53 ± 0.26 %
**Control cells**	0.52 ± 0.03 %

C.

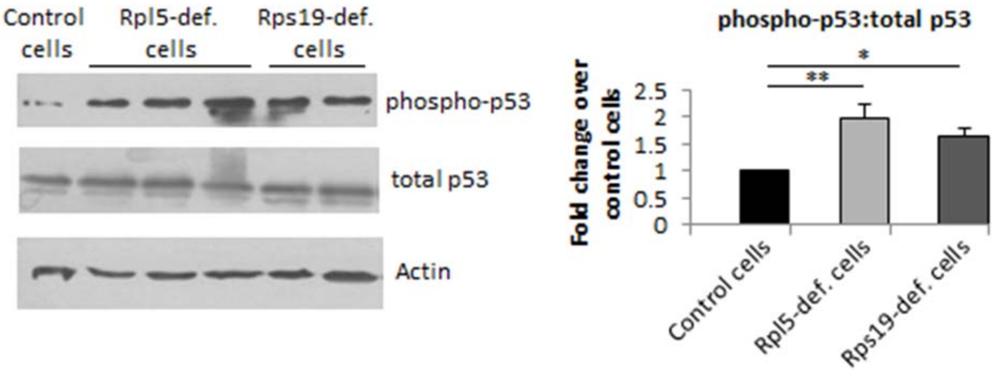

Figure S2: Characterization of Rpl5- and Rps19-deficient MEL cells

A. Proliferation assay. Significantly decreased proliferation capacity, at 48 and 72 hours, was detected for uninduced Rpl5- and Rps19-deficient cells in comparison with control cells using the MTT test. *P* values were calculated using the Student t-test; *** for $p < 0.001$. The experiment was repeated 4-times. **B. TUNEL assay.** Higher percentage of nucleated cells undergoing apoptosis ($DAPI^+/TUNEL^+$) was detected on cytospin slides of uninduced Rpl5- and Rps19-deficient cells in comparison with control cells using immunocytochemistry. The results represent the mean of 3 independent experiments. **C. Immunoblot analysis of phospho-p53 protein.** Increased level of p53 phosphorylation was detected in uninduced Rpl5- deficient cells (clones C1-C3) and Rps19-deficient cells (clone C1, C2) in comparison with control cells; phospho-p53 was normalized to total p53 protein. A representative immunoblot is shown. Data in the bar graph are expressed as means ± standard errors of the mean from 3 independent experiments. *$p < 0.05$ and **$p < 0.01$ versus control cells. ImageJ was used for densitometry [7].

Figure S3:

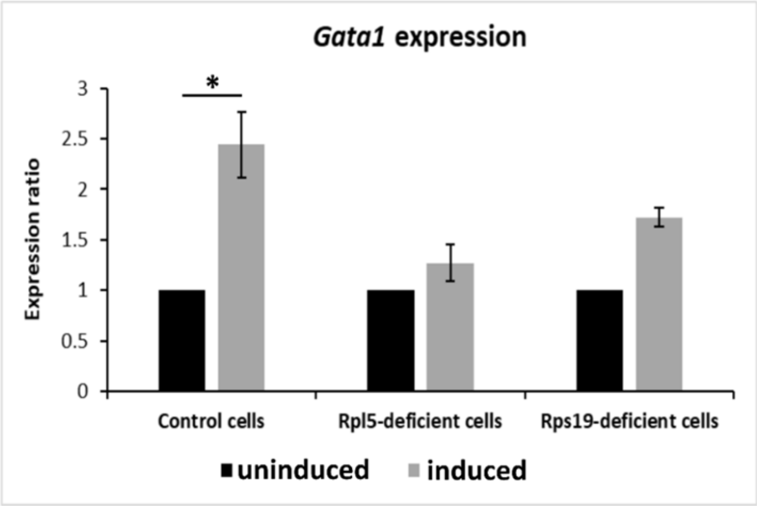

Figure S3: Relative expression of *Gata1*.

Gata1 mRNA expression (normalized to beta-actin) is substantially lower in Rpl5- and Rps19-deficient cells induced to erythroid differentiation (1.2-fold and 1.7-fold, respectively) when compared to control cells (2.4-fold). *P* values were calculated using the REST© 2009 software [6]; * for $p < 0.05$. The experiment was repeated 3 times.

Figure S4:

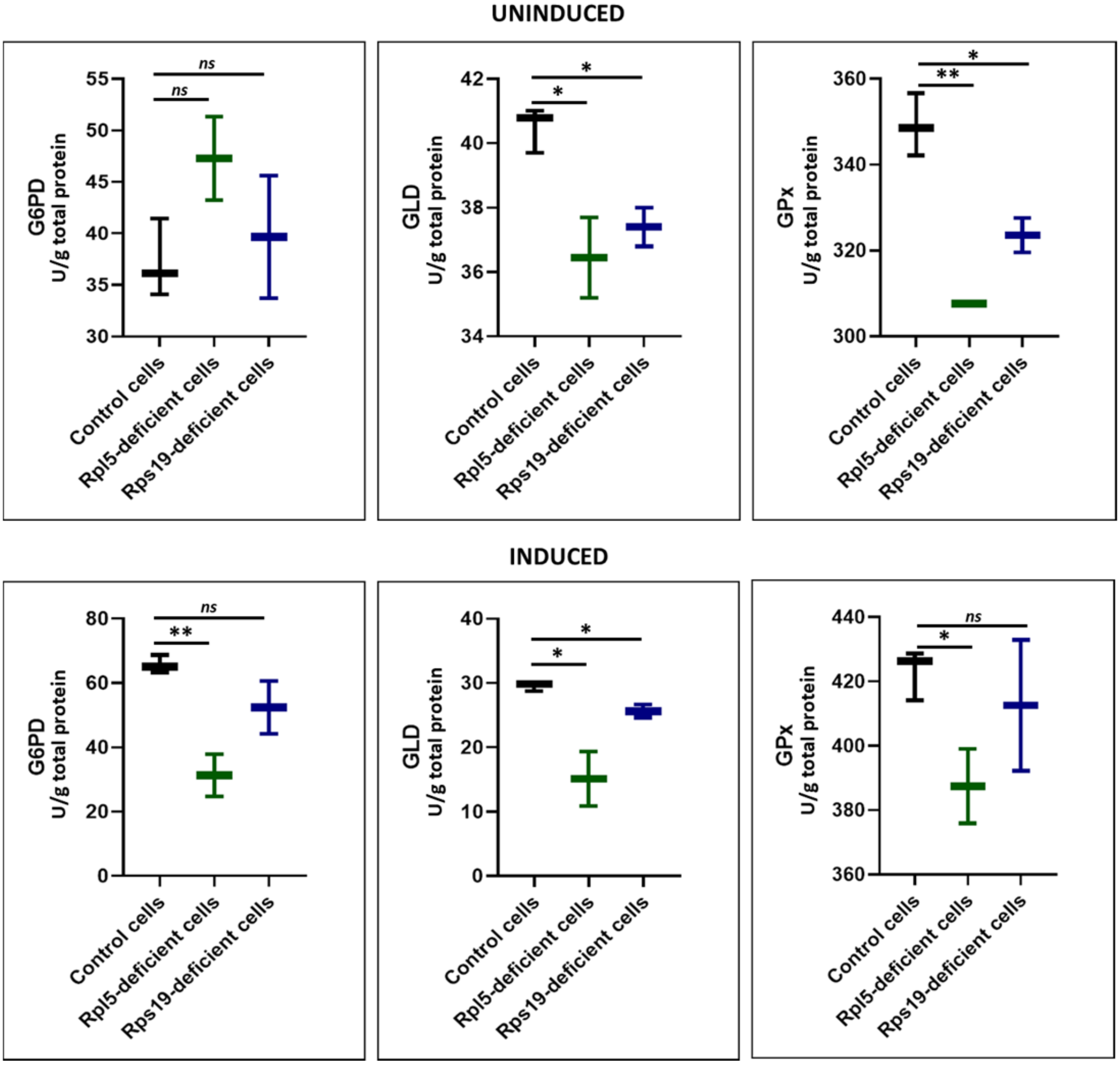

Figure S4: Activities of ROS scavenging enzymes in Rpl5- and Rps19-deficient cells.

Enzymes activities of G6PD, GLD, and GPx (expressed as U/g total protein) were either comparable to controls or significantly reduced. Values are presented as median (horizontal line) and the highest and the lowest values (the highest and the lowest horizontal lines, respectively) of 4 independent measurements. Specific enzyme activity was calculated using the Lambert-Beer law. Graphs and *p* values were calculated using GraphPad Prism 8 Software (La Jolla, CA, USA, www.graphpad.com); *$p < 0.05$, **$p < 0.01$, *ns* - not significant.

Figure S5:

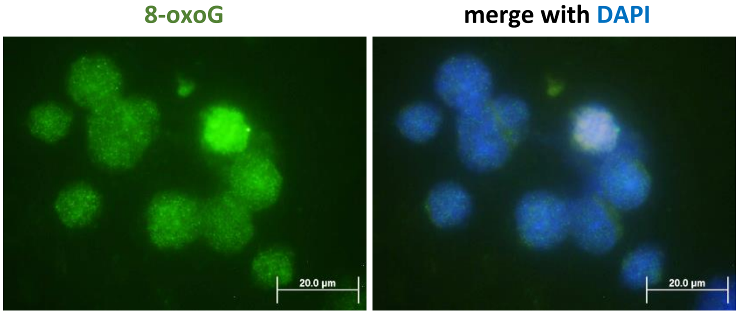

Figure S5: Positive controls for 8-oxoG staining.

8-oxoG positive control for ICC. To induce oxidative DNA damage, control MEL cells were treated with 100 µM hydrogen peroxide (H_2O_2) for 24 hours before staining; 8-oxoG positivity (green color), cell nuclei (DAPI, blue color).

Figure S6:

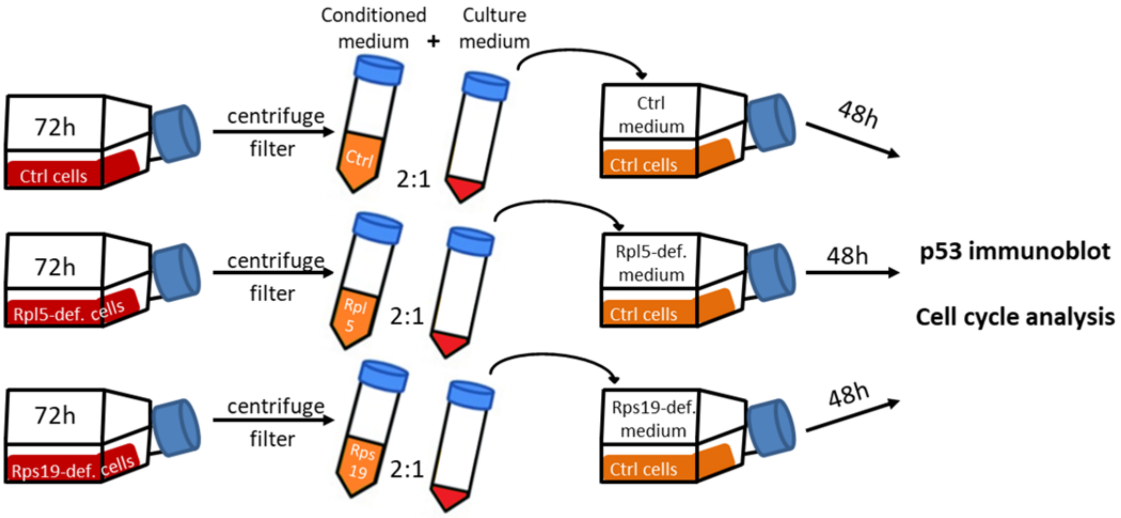

Figure S6: Effect of conditioned medium harvested from Rpl5- and Rps19-deficient cells on p53 activation and cell cycle of control cells.

Schematic diagram of the experimental procedure. The conditioned medium was obtained from control cells, Rpl5- and Rps19-deficient cells grown for 72 hours. Control cells were then cultivated for 48 hours in medium containing conditioned medium and normal culture medium in 2:1 ratio. The activation of p53 and cell cycle analyses were subsequently performed. Control cells' conditioned medium was used as a negative control; Ctrl cells – control cells.

Figure S7:

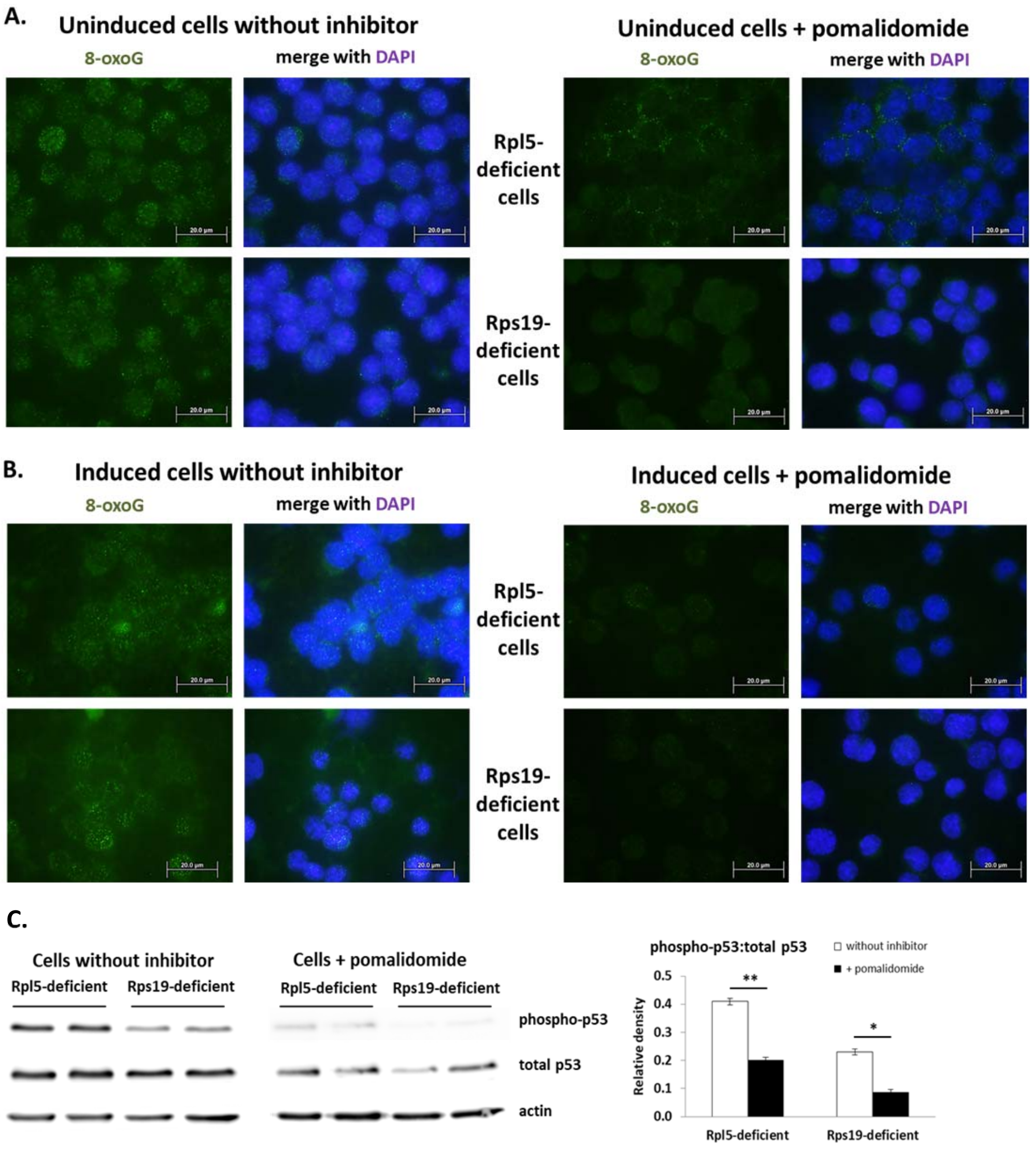

Figure S7: Immunocytochemistry staining for 8-oxoG and immunoblot analysis for phospho-p53 protein in Rpl5- and Rps19-deficient cells; the effect of pomalidomide.

Pomalidomide treatment (right panels) diminished nuclear and perinuclear 8-oxoG-positivity in uninduced (**A.**) and induced (**B.**) Rpl5- and Rps19-deficient cells. Representative images are shown. Immunostained cells were analyzed with an Olympus BX 51 fluorescence microscope (Olympus), original magnification 1000x. Digital images were acquired with an Olympus DP 50 camera driven by DP controller software (provided by Olympus). Images were cropped, assembled, and labeled using Adobe Photoshop software (Adobe Systems, San Jose, CA, USA). **C.** Pomalidomide treatment reduced p53 activation in uninduced Rpl5- and Rps19-deficient cells. Phospho-p53 was normalized to total

p53 protein level using the G:BOX-CHEMI-XX9 imaging system (Syngene, Cambridge, UK). The experiment was repeated 3 times.

Figure S8:

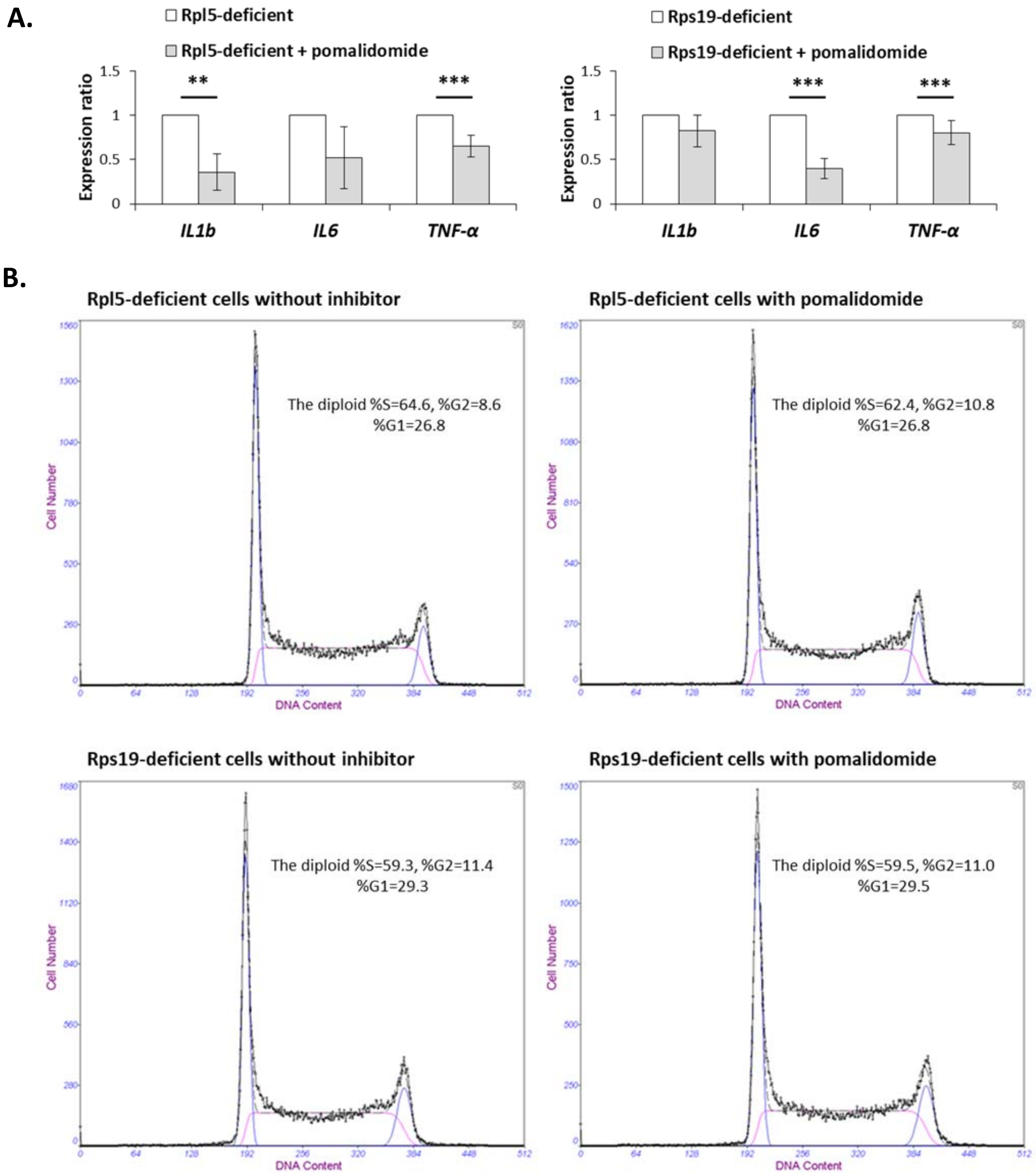

Figure S8: Effect of pomalidomide on the expression of inflammatory cytokines and cell cycle progression in uninduced Rpl5- and Rps19-deficient cells.

Pomalidomide treatment (for 48 h) suppressed mRNA expression of *IL1b, IL6, and TNF-α* (**A.**), but did not induce any changes in the cell cycle progression (**B.**) in Rpl5- and Rps19-deficient cells. *P* values were calculated using the REST© 2009 software [6]; ** for $p < 0.01$; *** for $p < 0.001$. Representative histograms are shown in Panel B. The experiment was repeated 3 times.

Figure S9:

A.

PCT	MUT	p53$^+$ cells [%]	TUNEL$^+$ cells [%]
P2	RPL5	5.9	13.0
P4	RPL5	8.4	17.6
P9	RPS19	9.8	13.3
P20	RPS19	7.3	18.5
P21	RPS19	3	22.8
P22	RPS26	1.9	3.3
P23	RPS26	6.9	9.9
P24	RPL11	13	7.3
Controls		*nd*	1.2±0.3

B. p53 expression

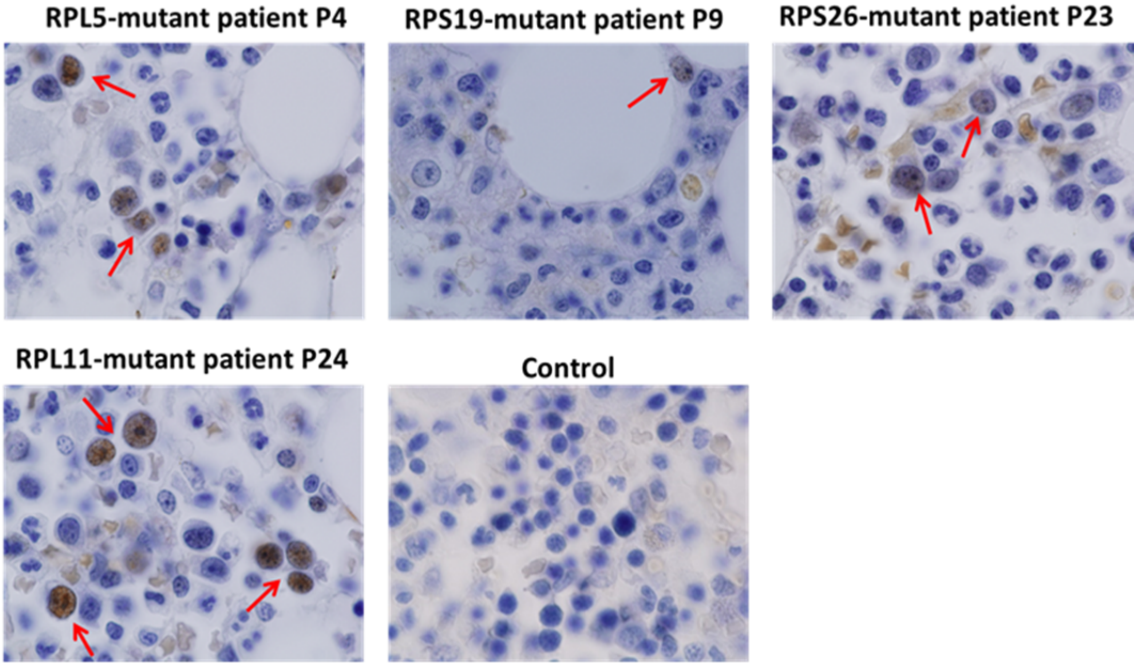

C. TUNEL assay

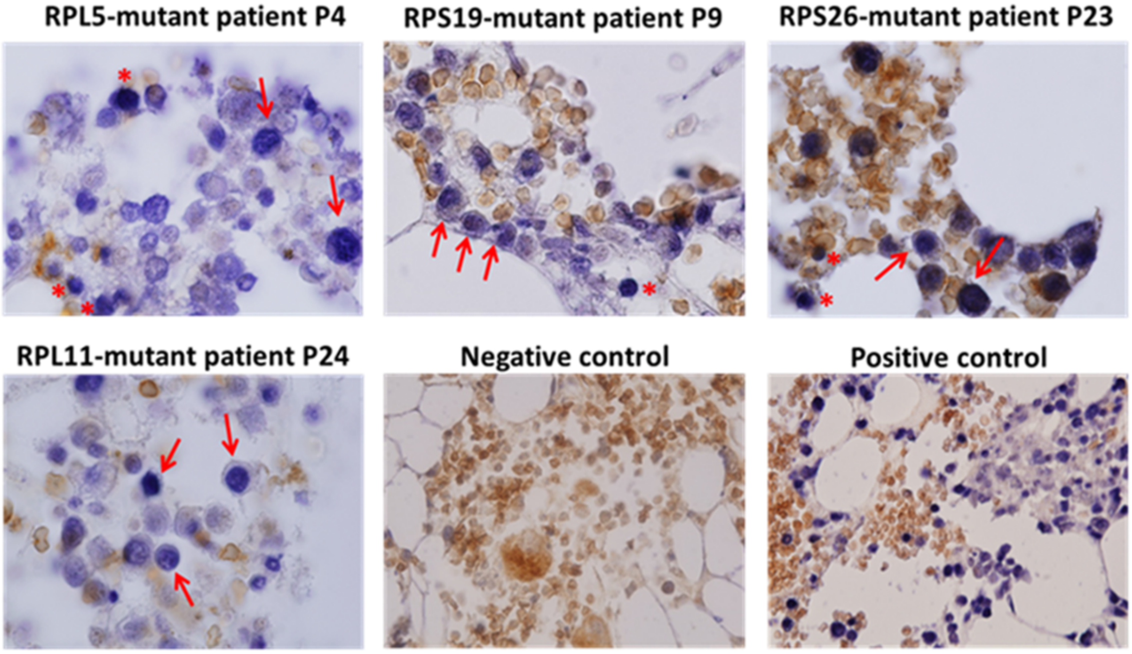

Figure S9. Immunohistochemical staining of bone marrow specimens.

A. Table showing percentage of p53 positive and TUNEL positive cells detected in individual patients' bone marrow trephine biopsy samples. *nd* not detected. **B. Evaluation of p53 expression in the bone marrow.** IHC staining revealed increased nuclear staining for p53 (brown color) in all analyzed DBA patients (Table 1) compared to controls. Cells were counterstained by hematoxylin (dark purple color). Positive cells belonging to granulocytic lineage (identified based on histopathological characteristics, i.e. cellular and nuclear morphology/lobulation and morphology of adjacent developing cells) are marked with an arrow. Representative images, one for each genotype, are shown; original magnification 1000x. **C. Assessment of apoptosis in the bone marrow.** Elevated numbers of cells undergoing apoptosis (TUNEL+, dark blue color) were detected in all analyzed DBA patients (Table 1) compared to controls. $TUNEL^+$ erythroblasts (double-stained with glycophorin A – light brown color) [8] are marked with an asterisk (*) and positive cells of granulocytic lineage are marked with an arrow. Positive control: slides were incubated with DNase I (10 U/mL) to induce DNA strand breaks prior to labeling procedure. Representative images, one for each genotype, are shown; original magnification 1000x (for DBA mutant patients) and 400x (for positive and negative control). The slides in both Panels were analyzed with an Olympus BX 51 light microscope (Olympus). Digital images were acquired with an Olympus DP 50 camera driven by DP controller software (provided by Olympus). Images were cropped, assembled, and labeled using Adobe Photoshop software (Adobe Systems).

Figure S10:

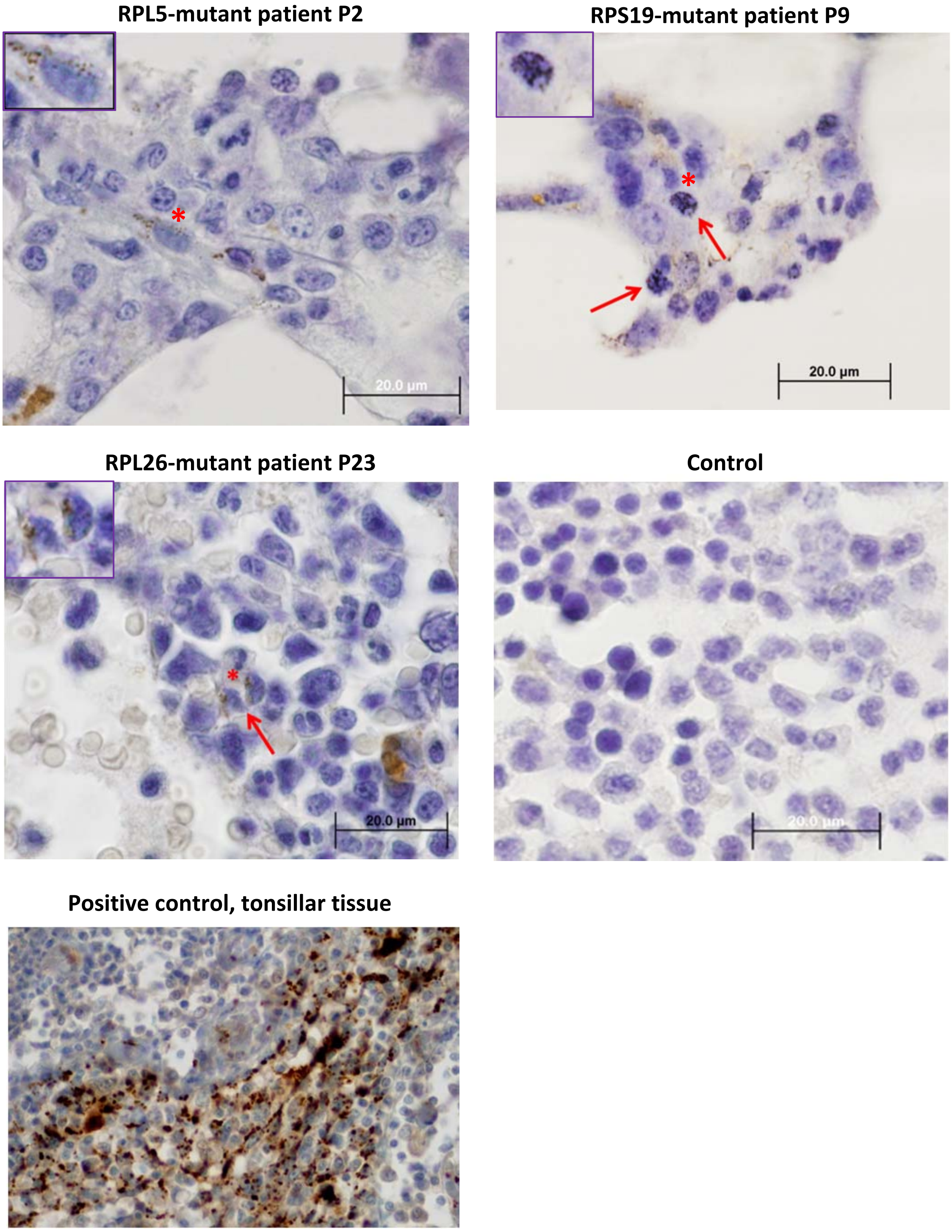

Figure S10. Immunohistochemistry for 8-oxoG in the bone marrow specimens.

Rare 8-oxoG-positive cells showing nuclear and perinuclear staining (brown color) were detected in DBA patients but not in controls. Cells were counterstained by hematoxylin (dark purple color). Representative images for given genotypes are shown. The arrows indicate positive cells of granulocytic lineage identified based on cellular and nuclear morphology and adjacent developing cells. The asterisks indicate cells shown in the insets. Tonsillar tissue was used as a positive control

for 8-oxoG staining. The slides were analyzed with an Olympus BX 51 light microscope (Olympus); original magnification 1000x. Digital images were acquired with an Olympus DP 50 camera driven by DP controller software (provided by Olympus). Images were cropped, assembled, and labeled using Adobe Photoshop software (Adobe Systems).

Figure S11:

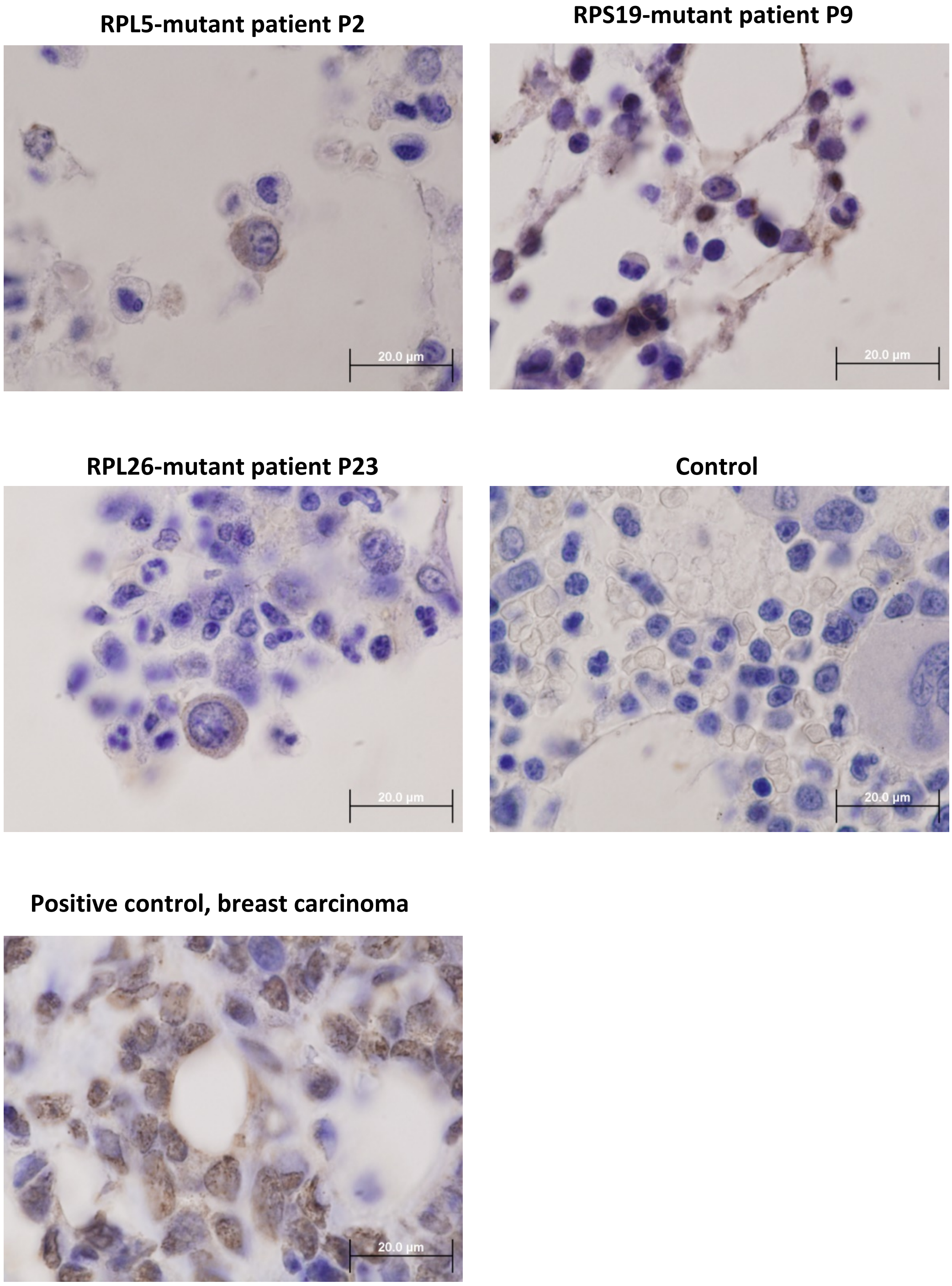

Figure S11. Immunohistochemistry for pATM in the bone marrow specimens.

Cytoplasmic positivity for phosphorylated ATM (pATM) at S1981 (brown color) was observed in sporadic cells in the bone marrow of DBA patients. Increased number of pATM positive cells, with both cytoplasmic and nuclear staining, was detected in one RPS19-mutant patient (P9). Cells were counterstained by hematoxylin (dark purple color). Representative images for given genotypes are shown. Breast carcinoma was used as a positive control for pATM staining. The slides were analyzed

with an Olympus BX 51 light microscope (Olympus); original magnification 1000x. Digital images were acquired with an Olympus DP 50 camera driven by DP controller software (provided by Olympus). Images were cropped, assembled, and labeled using Adobe Photoshop software (Adobe Systems).
